# Exploring the Molecular Mechanism of Comorbidity of Type 2 Diabetes Mellitus and Colorectal Cancer: Insights from Bulk Omics and Single-Cell Sequencing Validation

**DOI:** 10.3390/biom14060693

**Published:** 2024-06-14

**Authors:** Yongge Luo, Lei Yang, Han Wu, Hui Xu, Jin Peng, You Wang, Fuxiang Zhou

**Affiliations:** 1 Department of Radiation and Medical Oncology, Zhongnan Hospital of Wuhan University, Wuhan 430071, China; 2 Hubei Key Laboratory of Tumor Biological Behaviors, Wuhan 430071, China; 3 Hubei Provincial Clinical Research Center for Cancer, Wuhan 430071, China

**Keywords:** type 2 diabetes mellitus, colorectal cancer, comorbidity, EVPL, ENTPD3

## Abstract

The relationship between type 2 diabetes mellitus (T2DM) and colorectal cancer (CRC) has long been extensively recognized, but their crosstalk mechanisms based on gene regulation remain elusive. In our study, for the first time, bulk RNA-seq and single-cell RNA-seq data were used to explore the shared molecular mechanisms between T2DM and CRC. Moreover, Connectivity Map and molecular docking were employed to determine potential drugs targeting the candidate targets. Eight genes (*EVPL*, *TACSTD2*, *SOX4*, *ETV4*, *LY6E*, *MLXIPL*, *ENTPD3*, *UGP2*) were identified as characteristic comorbidity genes for T2DM and CRC, with *EVPL* and *ENTPD3* further identified as core comorbidity genes. Our results demonstrated that upregulation of EVPL and downregulation of ENTPD3 were intrinsic molecular features throughout T2DM and CRC and were significantly associated with immune responses, immune processes, and abnormal immune landscapes in both diseases. Single-cell analysis highlighted a cancer-associated fibroblast (CAF) subset that specifically expressed ENTPD3 in CRC, which exhibited high heterogeneity and unique tumor-suppressive features that were completely different from classical cancer-promoting CAFs. Furthermore, ENTPD3^+^ CAFs could notably predict immunotherapy response in CRC, holding promise to be an immunotherapy biomarker at the single-cell level. Finally, we identified that droperidol may be a novel drug simultaneously targeting EVPL and ENTPD3. In conclusion, previous studies have often focused solely on metabolic alterations common to T2DM and CRC. Our study establishes EVPL and ENTPD3 as characteristic molecules and immune biomarkers of comorbidity in T2DM and CRC patients, and emphasizes the importance of considering immunological mechanisms in the co-development of T2DM and CRC.

## 1. Introduction

Colorectal cancer (CRC) is the third most common cancer and the second most deadly cancer worldwide. As the most prevalent malignancy in the digestive system, CRC accounts for 1.88 million new cases and 920,000 deaths annually, and the disease burden continues to rise [1]. Approximately 15% of CRC cases are driven by mismatch repair defects (dMMR) [2]. Such patients have certain survival advantages and may benefit from immunotherapy. Unfortunately, the vast majority of CRC exhibits mismatch repair proficiency (pMMR), which is associated with more aggressive biological behavior and very limited treatment options. Therefore, the overall prognosis of CRC remains poor, underscoring the urgent need for a better understanding of CRC molecular pathogenesis to develop novel and effective treatment strategies.

Diabetes mellitus is the most common endocrine disease characterized by chronic hyperglycemia resulting from impaired insulin secretion and/or utilization [3]. According to the International Diabetes Federation, an estimated 537 million people were affected by diabetes mellitus worldwide in 2021, with more than 90% of them having type 2 diabetes mellitus (T2DM) [4]. In addition to causing severe vascular complications, T2DM is also a major contributor to cancer, especially digestive system cancers represented by CRC. Multiple previous studies have consistently revealed a remarkably higher risk of developing CRC in patients with T2DM than in normal individuals [5,6]. The presence of T2DM also reduces the overall survival of CRC patients [7,8]. A large cohort study involving 137,804 individuals showed a 2.4-fold increase in risk of CRC mortality among T2DM patients compared to the general population [9]. According to existing studies, possible causes of T2DM inducing CRC include hyperglycemia [10], insulin resistance and hyperinsulinemia [11,12], obesity and systemic inflammation [13], gastrointestinal dysmotility [14], and dysfunction of NK cells [15]. However, T2DM may also be a consequence of CRC. A significant increase in the risk of new-onset diabetes has been observed in CRC survivors [16]. Therefore, CRC and T2DM may share the same crosstalk mechanisms. Moreover, long-term use of exogenous insulin increases CRC risk among T2DM patients [17]. Conversely, T2DM patients treated with metformin or thiazolidinediones have reduced incidence rates of CRC [18,19], as these are classical drugs for improving insulin resistance. Despite strong clinical and epidemiological evidence indicating the interplay between T2DM and CRC, the shared mechanisms and key molecular features based on gene regulation are still largely unknown.

The rapid development of next-generation sequencing technology has greatly promoted human insight into disease pathogenesis at the gene level. Previous genome-wide gene-by-environment interaction study (GEWIS) showed that some genes, such as *SLC30A8* and *LRCH1*, were involved in the co-development of T2DM and CRC [20], but this only explained a small portion of the genetic factors. Liu et al. constructed a 14-gene model to predict the prognosis of CRC patients but did not explore the key genes and underlying mechanisms for the two diseases [21]. In comparison to traditional RNA sequencing (RNA-seq), single-cell RNA sequencing (scRNA-seq) defines the global gene expression profiles of individual cells, enabling a better understanding of tumor heterogeneity and a given tumor microenvironment (TME). For some previous research, scRNA-seq has emerged as the technology of choice for dissecting characteristics of various cell populations and intracellular/intercellular molecular mechanisms within CRC and normal adjacent tissues (NATs) [22]. In this study, we integrated bulk RNA-seq and scRNA-seq to identify central pathways and core genes involved in T2DM and CRC, aiming at elucidating the shared pathogenic mechanisms and molecular markers underlying the reciprocal crosstalk between the two diseases, which will facilitate better serving T2DM patients and providing early diagnostic and therapeutic strategies for CRC patients.

## 2. Materials and Methods

### 2.1. Download and Pre-Processing of Bulk RNA-Seq Data

Through systematic data retrieval strategies, the microarray datasets GSE20966, GSE25724, GSE50397, GSE184050, GSE41762, GSE32323, GSE68468, GSE25070, GSE113513, GSE24549, GSE143985, GSE33193, GSE41657, GSE192667, and GSE41258 on T2DM and CRC were downloaded from the Gene Expression Omnibus (GEO) database (https://www.ncbi.nlm.nih.gov/geo/, accessed on 8 March 2023) [23]. The high-throughput sequencing dataset TCGA-CRC was downloaded from The Cancer Genome Atlas (TCGA) database (https://portal.gdc.cancer.gov/, accessed on 15 March 2023) [24]. Details about each dataset are shown in Supplementary Table S1. Detailed information on the inclusion criteria for each dataset is provided in Supplementary Table S2. We conducted a series of pre-processing steps on the microarray expression profiles obtained from GEO, including background correction, normalization, and log2 transformation. This was accomplished using the “rma” function from the “affy” R package [25], the “lumiExpresso” function from the “lumi” R package [26], and the “backgroundCorrect” and “normalizeBetweenArrays” functions from the “limma” R package [27]. To ascertain the quality and consistency of the datasets, we employed rigorous exploratory data analysis techniques. Multiple data quality control methods were employed to validate the effectiveness of the data pre-processing methods, including generating box plots, principal component analysis (PCA) plots, and data quality control plots. Next, to enhance the interpretability and utility of the data, we utilized the GPL platform information for each dataset to convert the probe names of the microarray expression matrix into gene names. Particularly, during probe annotation for each GEO dataset, we removed individual probes that corresponded to multiple genes simultaneously. In cases where multiple probes corresponded to a single gene, we calculated the average expression value of all probes corresponding to that gene. For the TCGA-CRC cohort, all gene expression values were transformed into transcripts per million (TPM) and subsequently analyzed after log2 normalization.

### 2.2. Weighted Gene Co-Expression Network Analysis (WGCNA)

WGCNA is a systems biology methodology utilized to characterize gene correlation patterns among different samples. It facilitates the clustering of genes exhibiting highly coordinated changes and identifies potential biomarker genes or therapeutic targets based on the interconnectedness of gene sets and their associations with phenotypes [28]. In numerous rigorous scientific studies, WGCNA has been extensively used to exploit the intricate relationship between gene expression profiles and phenotypes. In this study, we used the R package “WGCNA” [28] to construct weighted gene co-expression networks of T2DM and CRC. Our construction process primarily includes the following steps: 1. Calculating the soft threshold β using the “pickSoftThreshold” function; 2. Computing the adjacency matrix based on the soft threshold using the “adjacency” function and converting it into a topological overlap matrix with the “TOMsimilarity” function. 3. Generating and cutting the hierarchical clustering tree to identify gene modules utilizing the “hclust” function and “cutreeDynamic” functions (with minClusterSize = 50); 4. Calculating module eigengenes using the “moduleEigengenes” function; 5. Merging similar modules with the “mergeCloseModules” function; 6. Conducting gene-group correlation analysis using the “cor” and “corPvalueStudent” functions.

### 2.3. Identification of Differentially Expressed Genes (DEGs)

The “limma” R package was used to calculate DEGs for the classic T2DM (GSE25724) and CRC (GSE68468) datasets. This included creating design matrixes describing sample information using the “model.matrix” function, fitting a linear model for each gene across all samples using the “lmFit” function, optimizing variance estimation in differential expression analysis using the “eBayes” function, and performing multiple testing correction based on the false discovery rate (FDR) method using the “topTable” function to extract significant differentially expressed genes. Significant DEGs were screened based on adjusted *p*-value < 0.05 and |log2FC| ≥ 0.6, which ensured the robustness of the identification of statistically differentially expressed genes.

### 2.4. Functional Enrichment Analysis

Enrichment analysis is a statistical method used to determine whether a specific set of genes or proteins shows a statistically significant, non-random association with particular biological pathways or processes, thereby linking experimental data with biological significance. Gene Ontology (GO) enrichment analysis identifies the functions of genes or gene products across three aspects: cellular components (CC), molecular functions (MF), and biological processes (BP) [29]. Kyoto Encyclopedia of Genes and Genomes (KEGG) enrichment analysis focuses on the functional pathways that genes or gene products are involved in [30]. Gene Set Enrichment Analysis (GSEA) is used to determine if a predefined set of genes shows statistically significant, consistent differences between two biological states [31]. To explore the potential biological mechanisms of the identified genes, we performed GO/KEGG enrichment analysis and GSEA. Specifically, after converting IDs using the “org.Hs.eg.db” R package (https://bioconductor.org/packages/release/data/annotation/html/org.Hs.eg.db.html, accessed on 22 April 2023), GO and KEGG enrichment analyses were conducted using the “enrichGO” and “enrichKEGG” functions from the “clusterProfiler” R package [32]. For GSEA, we downloaded the gmt file (c2.cp.all.v2022.1.Hs.symbols.gmt, accessed on 10 November 2022) of the marker gene sets from MSigDB (https://www.gsea-msigdb.org/gsea/msigdb, accessed on 10 November 2022) [33] and executed GSEA using the “GSEA” function from the R package “clusterProfiler”.

### 2.5. Immune Landscape Assessment and Immunotherapy Response Prediction

The single-sample Gene Set Enrichment Analysis (ssGSEA) immune infiltration algorithm uses specific markers for each type of immune cell as gene sets to calculate enrichment scores for each type of immune cell in individual samples, thus inferring the infiltration of immune cells in each sample [34]. Using the “gsva” function from the “GSVA” R package [35], we carried out ssGSEA for abundance measurement of each immune cell type in diabetic samples from the T2DM datasets (GSE184050 and GSE41762) and tumor samples from the TCGA-CRC cohort. The specific markers for the 24 types of immune cells used are sourced from previous research [36].

The ESTIMATE algorithm uses transcriptome expression profiles to infer the abundance of immune cells and stromal cells in the TME, thereby predicting tumor purity [37]. We utilized the “estimateScore” function of the “estimate” R package to calculate the StromalScore (represents the infiltration level of stromal cells), ImmuneScore (represents the infiltration level of immune cells), and ESTIMATEScore (represents the infiltration level of non-tumor cells) for each tumor sample in the TCGA-CRC cohort. Tumor purity was calculated using the given formula:

$$Tumor\;purity=cos\;\left(0.6049872018+0.0001467884\times ESTIMATEScore\right)$$

Subsequently, 5 external immunotherapy cohorts (Gide et al. (anti-PD-1) [38], Gide et al. (anti-PD-1+anti-CTLA-4) [38], Kim et al. (anti-PD-1) [39], IMvigor210 (anti-PD-L1) [40], and Braun et al. (anti-PD-1) [41]) were used to examine the relationship between EVPL and ENTPD3 and cancer immunotherapy. Details about each dataset are shown in Supplementary Table S3. Detailed information on the inclusion criteria for each dataset is provided in Supplementary Table S4.

### 2.6. Integration and Processing of scRNA-Seq Data

We included 52 single-cell sequencing samples from 31 CRC patients at different treatment and pre-biopsy statuses, comprising 25 primary CRC/matched NAT samples from 12 patients who underwent initial surgery without prior treatment (GSE166555) and 27 primary CRC samples from 19 patients before and after neoadjuvant immunotherapy (GSE205506). Details about each dataset are shown in Supplementary Tables S5–S7. Detailed information on the inclusion criteria for each dataset is provided in Supplementary Table S8. All scRNA-seq data analysis was based on the R package “Seurat” [42]. First, we excluded genes expressed in <3 cells, cells expressing <250 genes, and potential doublets and multiplets. Considering the high metabolic activity in colon tissue [43], cells with a mitochondrial gene proportion <20% in NATs and <30% in CRC were selected for subsequent analysis. We conducted data quality control to ensure that our data filtering helps achieve a balance between identifying vital results and minimizing the risk of incorrect major results. Next, a range of downstream data processing steps were conducted, including normalization (“NormalizeData” function), finding highly variable genes (“FindVariableFeatures” function), scaling (“ScaleData” function), principal component analysis downscaling (“RunPCA” function), cell clustering (“FindNeighbors” and “FindClusters” function), and t-SNE non-linear downscaling (“RunTSNE” function). Batch effects between samples were removed using the “RunHarmony” function of the “harmony” R package. Finally, we manually annotated each cell subpopulation using known cellular markers.

### 2.7. Pseudotime Analysis

Pseudotime analysis constructs cell lineage development based on temporal changes in gene expression within different cell subsets in a given cell type. In this study, we reconstructed the differentiation trajectory of cancer-associated fibroblasts (CAFs) in CRC using the “monocle” R package [44] to delineate the sequential transition and evolution among distinct CAF subsets. Trajectory analysis mainly involved constructing the cds object (“newCellDataSet” function), identifying differentially expressed genes (“differentialGeneTest” function), dimensionality reduction (“reduceDimension” function), and cell ordering (“orderCells” function).

### 2.8. Connectivity Map (CMap) Drug Identification

CMap is a bioinformatics tool that uses gene expression data to explore interactions between drugs, genes, and diseases and is a key component of pharmacogenomics [45]. The principle of CMap is based on a core assumption: if the gene expression pattern under a certain biological condition (e.g., a disease state, a gene knockout, etc.) can be reversed by the gene expression pattern of a certain drug treatment, then the drug may have therapeutic effects on that condition. In this study, we used the next-generation CMap Library of Integrated Network-Based Cellular Signatures (LINCS) [46] for potential drug identification targeting EVPL and ENTPD3 in colorectal cancer. In detail, we utilized the “Discover LINCS Data” module of the LINCS online website (https://lincsproject.org/, accessed on 8 March 2023) to obtain the “Signature Similarity Search Results” for EVPL and ENTPD3. From the “LINCS L1000 Chemical Perturbations (2021)” result module, we downloaded all candidate Reversers and filtered them based on tissue type (intestine) and disease type (colon adenocarcinoma). Ultimately, 9 candidate reversers (droperidol, LFM-A13, indacaterol, amiodarone, zolmitriptan, levocetirizine, tipifarnib-P2, BRD-K78687850, and AS-703026) that may simultaneously reverse the upregulation of EVPL and downregulation of ENTPD3 in colorectal cancer were identified for further molecular docking analysis.

### 2.9. Molecular Docking Analysis

Molecular docking analysis was performed to explore the druggable potential of the two target genes for the candidate compounds. First, the two-dimensional structures of the candidate compounds were procured from the PubChem database [47] and transformed into three-dimensional conformations using Chem3D (https://library.bath.ac.uk/chemistry-software/chem3d, accessed on 12 November 2023). The three-dimensional protein structures of the target genes *EVPL* and *ENTPD3* were acquired respectively from the Protein Data Bank (PDB) [48] and the SWISS-MODEL repository (https://swissmodel.expasy.org/, accessed on 12 November 2023). After modifications to the target proteins, including removing water molecules, hydrogenation, and atom type setting, the AutoDock-Vina software (version 1.5.6) [49] was employed for molecular docking to evaluate the binding affinity of the receptor–ligand complexes. An affinity value of <−5 kcal/mol implies good binding stability and binding interactions between the compound and the target protein [50]. Lastly, the molecular conformations and binding modes of the receptor–ligand complexes were three-dimensionally visualized using PyMOL (https://www.pymol.org/, accessed on 8 March 2023).

### 2.10. Statistical Analysis

All statistical analyses were performed using R software (version 4.2.2). Receiver operating characteristic (ROC) curves were generated using the “roc” function of the R package “pROC” [51]. Survival analysis was conducted using Kaplan–Meier analysis [52] and the log-rank test. Differences between two independent continuous variables was assessed using the Student’s t-test or Wilcoxon rank-sum test. The paired t-test was employed to evaluate differences between two paired continuous variables. Differences among three or more continuous variables were analyzed using one-way ANOVA or the Kruskal–Wallis test. Differences in categorical variables between two groups were compared using the chi-square test. Pearson’s test or Spearman’s test was used to evaluate the correlation between two variables. *p*-values < 0.05 (two-tailed) were considered statistically significant.

## 3. Results

### 3.1. Co-Expression Modules in T2DM and CRC

**Figure 1:**
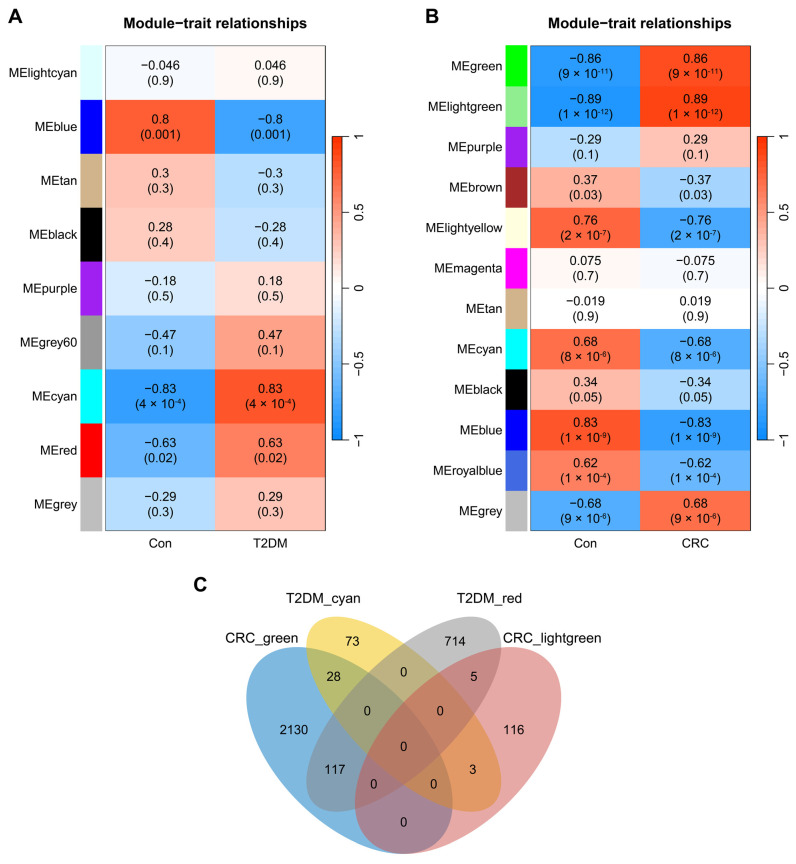
Identification of modules linked to clinical features of T2DM and CRC via WGCNA. (**A**) Heatmap of module–trait relationships in T2DM. (**B**) Heatmap of module–trait relationships in CRC. (**C**) Venn diagram of the shared genes between the two T2DM modules and two CRC modules.

Through WGCNA, we identified 9 modules in GSE20966. The “cyan” module (cor = 0.83, *p* = 4 × 10^−4^, genes = 104) and the “red” module (cor = 0.63, *p* = 0.02, genes = 836) were core modules highly positively associated with T2DM (Figure 1A). Moving on to the dataset GSE32323, we recognized 12 CRC-related modules. The “lightgreen” module and the “green” module were determined as the core modules most positively associated with CRC (green module: cor = 0.86, *p* = 9 × 10^−11^, genes = 2275; lightgreen module: cor = 0.89, *p* = 1 × 10^−12^, genes = 124; Figure 1B). By intersecting the four T2DM and CRC core modules, we successfully identified 153 core module genes that play a collective role in the regulatory processes of both T2DM and CRC (Figure 1C).

### 3.2. Identification of DEGs in T2DM and CRC

**Figure 2:**
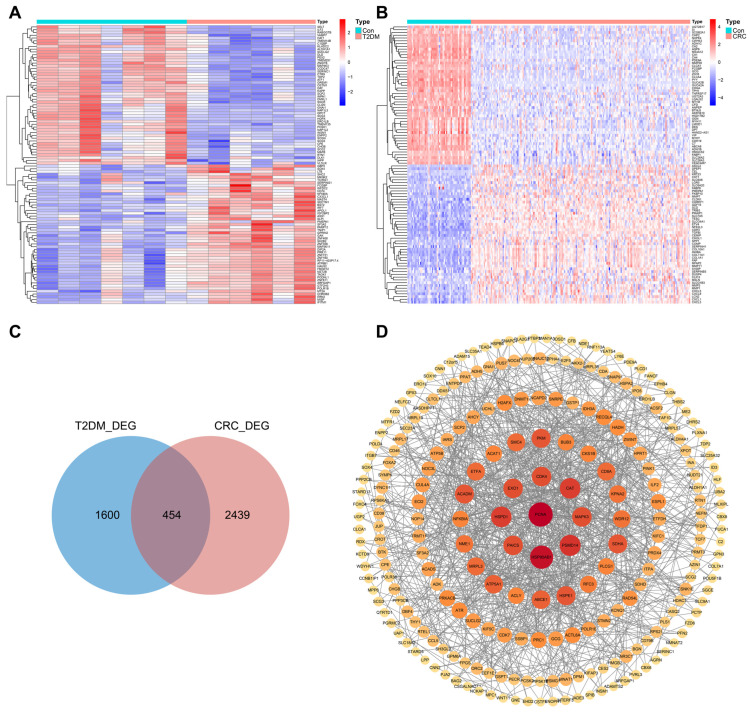
Verification and analysis of shared DEGs between T2DM and CRC. (**A**) Heatmap of the top 100 DEGs in T2DM. (**B**) Heatmap of the top 100 DEGs in CRC. (**C**) Venn diagram of the shared DEGs between T2DM and CRC. (**D**) PPI network of the shared DEGs (Proteins that interact with at least 3 proteins were displayed).

To ensure the accuracy of the shared genes ultimately identified, we calculated the DEGs of the GSE25724 and GSE68468 datasets (Figure 2A,B). By crossing the DEGs of the two datasets, we obtained 454 genes that were simultaneously differentially expressed in T2DM and CRC (Figure 2C). The STRING database (https://string-db.org/, accessed on 13 April 2023) was used to construct a protein–protein interaction (PPI) network to display the interaction relationships among the shared DEGs (Figure 2D). Next, we performed GO and KEGG enrichment analysis to explore the potential biological functions of the shared DEGs and found that they were significantly enriched in diverse metabolic and immune activities. GO analysis revealed their involvement in biological processes such as fatty acid metabolism, nucleotide metabolism, and immune regulation of myeloid lymphocytes (Supplementary Figure S1A). KEGG analysis suggested that the shared DEGs mainly engaged in pathways including carbon metabolism, pathways in cancer, pancreatic secretion, and T cell receptor signaling (Supplementary Figure S1B). Finally, we narrowed down the selection of DEGs by conducting a second intersection of the 454 DEGs, resulting in 267 core DEGs that exhibited the same differential expression trend in T2DM and CRC (Supplementary Figure S1C).

### 3.3. EVPL and ENTPD3 Are the Optimal Shared Genes

**Figure 3:**
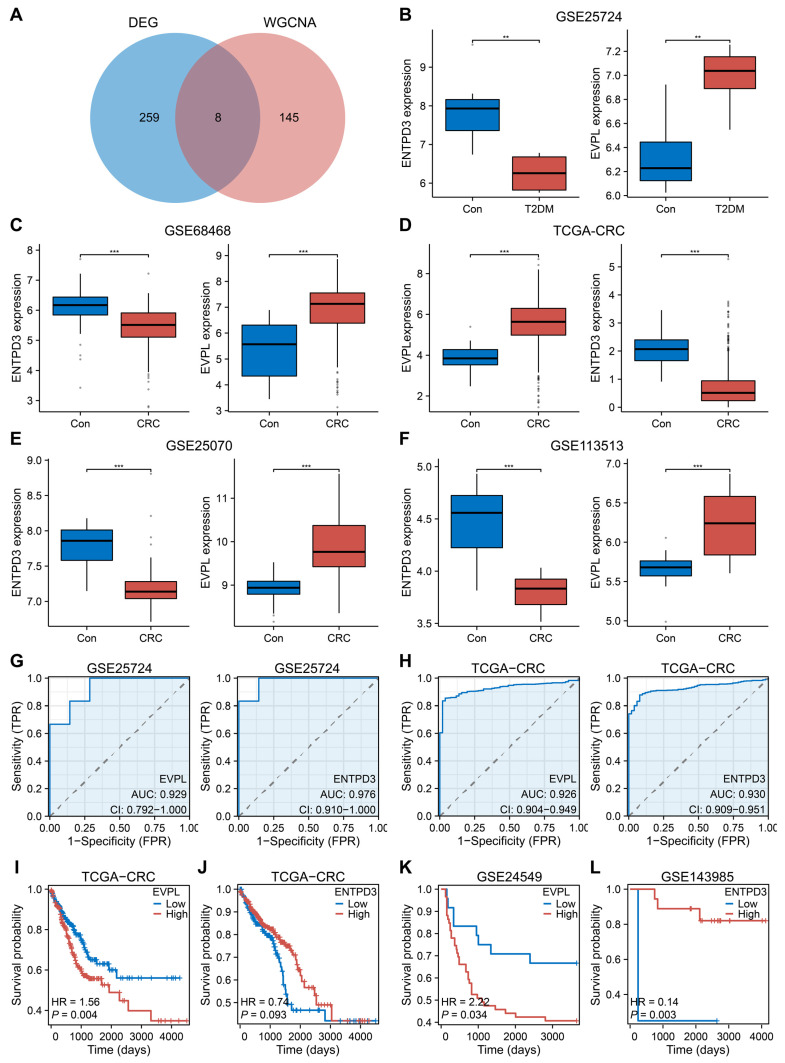
The expression, diagnostic, and prognostic value of EVPL and ENTPD3 in T2DM and CRC patients. (**A**) Venn diagram of the shared genes from the WGCNA and DEG analysis. (**B**) The expression of EVPL and ENTPD3 in pancreas tissues of T2DM compared with controls in the GSE25724 dataset. (**C**–**F**) The expression of EVPL and ENTPD3 in colon/colorectal tissues of CRC compared with controls in the GSE68468 (**C**), TCGA-CRC (**D**), GSE25070 (**E**), and GSE113513 (**F**) datasets. (**G**,**H**) ROC curve of EVPL and ENTPD3 in the GSE25724 dataset (**G**) and the TCGA-CRC cohort (**H**). (**I**) K–M curve of association of EVPL and CRC patients’ PFI in the TCGA-CRC cohort. (**J**) K–M curve of association of ENTPD3 and CRC patients’ OS in the TCGA-CRC cohort. (**K**) K–M curve of association of EVPL and CRC patients’ DFS in the GSE24549 dataset. (**L**) K–M curve of association of ENTPD3 and colon cancer patients’ DFS who have received adjuvant chemotherapy in the GSE143985 dataset. K–M, Kaplan–Meier; PFI, progress-free interval; OS, overall survival; DFS, disease-free survival. ** *p* < 0.01, and *** *p* < 0.001.

After combining 153 core module genes and 267 core DEGs, we derived eight potential core shared genes (*EVPL*, *TACSTD2*, *SOX4*, *ETV4*, *LY6E*, *MLXIPL*, *ENTPD3*, *UGP2*; Figure 3A). Considering the expression, prognosis, clinical value, and biological characteristics of the eight genes in both diseases, we ultimately determined *EVPL* and *ENTPD3* to be the optimal shared genes in the biological context of T2DM and CRC. Multiple internal and external datasets demonstrated that EVPL was significantly upregulated in T2DM and CRC, while ENTPD3 was significantly downregulated at the same time (Figure 3B–F). ROC curves further revealed their impressive diagnostic value in both diseases (EVPL for T2DM: area under the curve (AUC) = 0.929, ENTPD3 for T2DM: AUC = 0.976, EVPL for CRC: AUC = 0.926, and ENTPD3 for CRC: AUC = 0.930; Figure 3G,H). In terms of prognosis, as EVPL expression increased or ENTPD3 expression decreased, the survival of CRC patients became dramatically shorter (Figure 3I–L). Next, based on a series of clinical and clinicopathological parameters, we evaluated the clinical implications of aberrant expression of EVPL and ENTPD3 in T2DM and CRC patients. Glycosylated hemoglobin (HbA1c) is known to be an essential indicator in assessing diabetes control and also directly reflects the severity of diabetes mellitus [53]. We found that the HbA1c levels of T2DM patients were consistently elevated as EVPL expression increased or ENTPD3 expression decreased (Supplementary Figure S2A). For patients with CRC, high EVPL was predictive of worse histological grades, but high ENTPD3 had the opposite effect, suggesting a potential association between the core genes and the malignant biological behavior in CRC (Supplementary Figure S2B). As a classical precancerous lesion, colorectal adenoma has become an important target and surrogate endpoint for CRC screening. We noted that EVPL and ENTPD3 were already abnormally expressed at the adenoma stage compared to normal colorectal mucosa, and when colorectal adenoma progressed to colorectal adenocarcinoma, ENTPD3 expression was further downregulated (Supplementary Figure S2C). Hence, for T2DM patients, abnormalities of EVPL and ENTPD3 not only meant poor glycemic control but also simultaneously implied an increased risk of developing CRC. Going further, when localized CRC progressed to metastatic CRC, EVPL expression was further elevated, and ENTPD3 expression continually decreased, and ended up with a very low detection level (Supplementary Figure S2D). The analysis of LM showed the same tendency (Supplementary Figure S2E).

### 3.4. Biological Processes Associated with EVPL and ENTPD3

The GeneMANIA database (http://genemania.org/, accessed on 3 May 2023) was used to explore the underlying mechanisms of EVPL and ENTPD3. We identified 20 genes that are functionally related to *EVPL* and *ENTPD3* and used this information to construct a gene interaction network (Supplementary Figure S3A). GO analysis of the network genes revealed that *EVPL* primarily played a role in epithelial cell adhesion and migration and concurrently negatively regulated the migration of monocytes and lymphocytes. *ENTPD3* participated in nucleotide and energy metabolism and was also associated with T cell differentiation, regulation of regulatory T cell (Treg) differentiation, and T helper (Th) 17 type immune response (Supplementary Figure S3B).

### 3.5. Abnormal Expression of EVPL and ENTPD3 Predicts Altered Immune Landscape in T2DM and CRC

**Figure 4:**
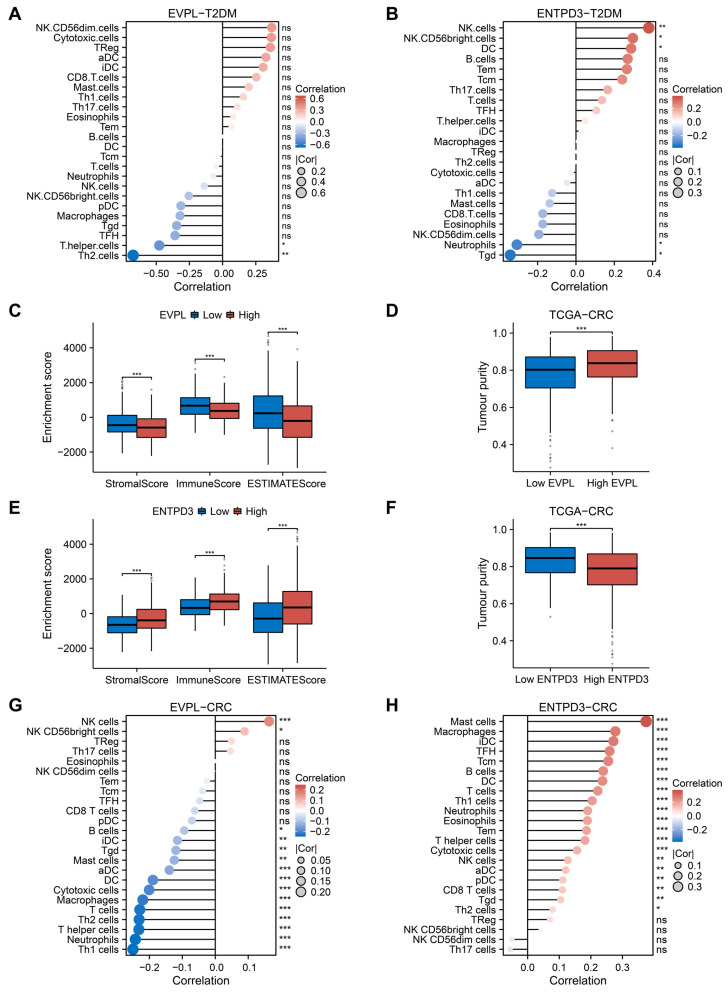
Immune landscape in T2DM and CRC patients with high and low expression of EVPL and ENTPD3. (**A**,**B**) Relationship between the expression of EVPL (**A**) and ENTPD3 (**B**) and immune cell subsets in T2DM patients using the ssGSEA algorithm. (**C**,**E**) Comparison of Stromalscore, Immunescore, and ESTIMATEScore between high and low expression groups of EVPL (**C**) and ENTPD3 (**E**). (**D**,**F**) Comparison of tumor purity between high and low expression groups of EVPL (**D**) and ENTPD3 (**F**). (**G**,**H**) Relationship between the expression of EVPL (**G**) and ENTPD3 (**H**) and immune cell subsets in CRC patients using the ssGSEA algorithm. ns, no significance, * *p* < 0.05, ** *p* < 0.01, and *** *p* < 0.001.

Both EVPL and ENTPD3 were enriched in the regulation of immune cells and immune response, implying that immune dysfunction related to EVPL and ENTPD3 may play a crucial role in the co-development of T2DM and CRC. Using the ssGSEA algorithm, we calculated the abundance of 24 immune cell types in diabetic samples from the T2DM datasets (GSE184050 and GSE41762) and tumor samples from the TCGA-CRC cohort. Next, we analyzed the relationship between the expression of EVPL and ENTPD3 and the infiltration levels of each immune cell type in samples of both diseases. We perceived that for T2DM patients, EVPL expression was negatively correlated with the infiltration level of Th cells and Th2 cells (Figure 4A). ENTPD3 expression was positively correlated with the infiltration of natural killer (NK) cells, CD56 bright NK cells, and dendritic cells (DCs), while negatively correlated with the infiltration of neutrophils and gamma delta T cells (Tgds) (Figure 4B). Furthermore, we used the ESTIMATE algorithm to calculate the StromalScore, ImmuneScore, ESTIMATEScore, and tumor purity of CRC samples in the TCGA-CRC cohort and compared the differences in these ESTIMATE scores between the high and low expression groups of EVPL and ENTPD3. The results showed that the high EVPL expression group had noticeably lower StromalScore, ImmuneScore, and ESTIMATEScore but higher tumor purity (Figure 4C,D). The low ENTPD3 expression group showed a completely homogeneous immune landscape with considerably high tumor purity and low infiltration of stromal cells and immune cells (Figure 4E,F). We further explored which immune cell types in CRC might be affected by the expression of EVPL and ENTPD3. Immune cell infiltration analysis based on the ssGSEA algorithm indicated that the expression of EVPL was positively correlated only with NK cell and CD56 bright NK cell infiltration, while negatively correlated with the infiltration of almost all other immune cells (Figure 4G). Conversely, but not surprisingly, ENTPD3 expression was positively correlated with the infiltration of almost all types of immune cells (Figure 4H).

### 3.6. EVPL and ENTPD3 Are Predictive Biomarkers for Cancer Immunotherapy

**Figure 5:**
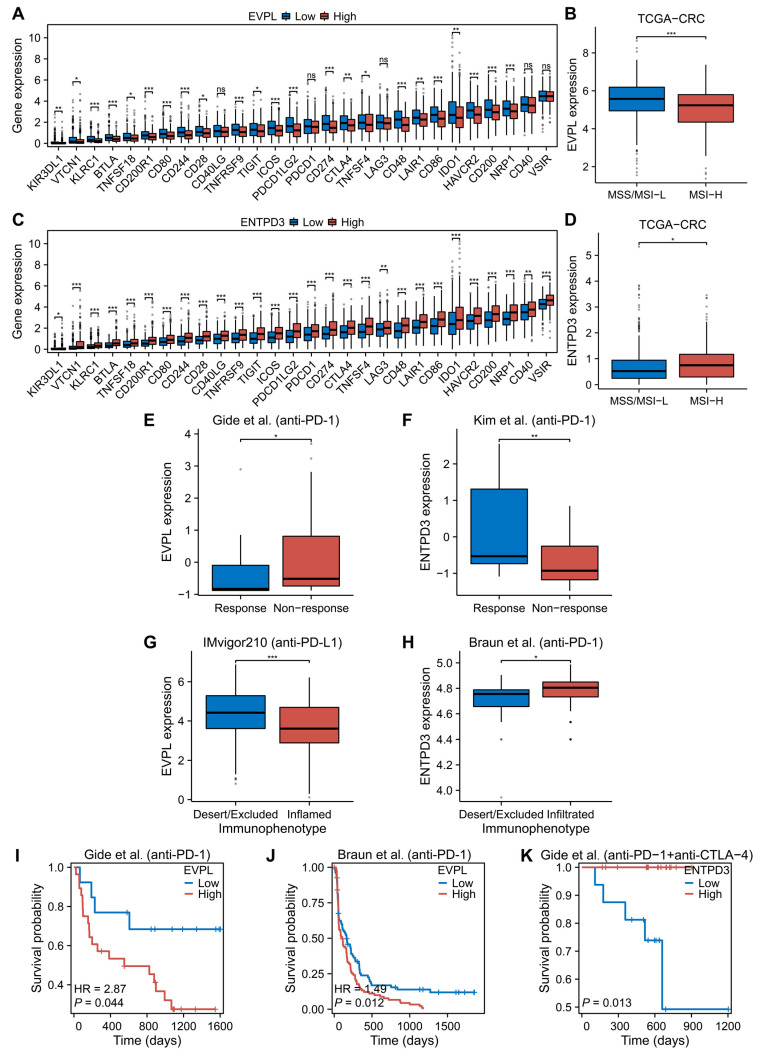
Association between EVPL and ENTPD3 expression and cancer immunotherapy. (**A**,**C**) Immune checkpoints between high and low expression groups of EVPL (**A**) and ENTPD3 (**C**) in CRC patients. (**B**,**D**) The expression of EVPL (**A**) and ENTPD3 (**C**) in CRC patients with different microsatellite instability (MSI) status. (**E**) Relationship between EVPL expression and immunotherapy response in melanoma patients [38]. (**F**) Relationship between ENTPD3 expression and immunotherapy response in gastric cancer patients [39]. (**G**) Relationship between EVPL expression and immunophenotype in urothelial cancer patients [40]. (**H**) Relationship between ENTPD3 expression and immunophenotype in clear-cell renal cell carcinoma patients [41]. (**I**) K–M curve of association of EVPL and melanoma patients’ OS in the Gide et al. (anti-PD-1) cohort [38]. (**J**) K–M curve of association of EVPL and clear-cell renal cell carcinoma patients’ PFS in the Branu et al. (anti-PD-1) cohort [41]. (**K**) K–M curve of association of ENTPD3 and melanoma patients’ OS in the Gide et al. (anti-PD-1+anti-CTLA-4) cohort [38]. PFS, progression-free survival. * *p* < 0.05, ** *p* < 0.01, and *** *p* < 0.001.

After ascertaining the relationship between EVPL and ENTPD3 and immune cell infiltration in T2DM and CRC, we continued to investigate their co-expression association with immune checkpoints in CRC. We compared the differences in expression of various immune checkpoints in high and low expression groups of EVPL and ENTPD3 in the TCGA-CRC cohort. Similar to the characteristics of immune cell infiltration, both the high EVPL expression group and the low ENTPD3 expression group markedly lacked immune checkpoint expression (Figure 5A,C). Furthermore, we observed that CRC with high EVPL expression predominantly manifested microsatellite stable/microsatellite instability-low (MSS/MSI-L), while those with high ENTPD3 expression exhibited microsatellite instability-high (MSI-H) (Figure 5B,D). Considering that EVPL and ENTPD3 were closely related to immune cell infiltration, immune checkpoints, and microsatellite instability (MSI) status of CRC, we speculated that EVPL and ENTPD3 may serve as predictive biomarkers for CRC immunotherapy. Due to the dearth of sufficient available data on CRC immunotherapy, we first investigated the ability of EVPL and ENTPD3 expression to predict immunotherapy response in other cancer types. We compared the expression levels of EVPL and ENTPD3 in multiple immunotherapy cohorts of patients with different immunotherapy responses and immunophenotypes. As would be expected, high EVPL expression implied a suboptimal response to nivolumab and pembrolizumab in melanoma (Figure 5E), while gastric cancer patients with high ENTPD3 expression responded visibly better to pembrolizumab (Figure 5F). We further found that urothelial cancer with high EVPL expression presented as an immune-excluded or immune-desert phenotype, while clear-cell renal cell carcinoma with high ENTPD3 expression leaned to be immune-inflamed (Figure 5G,H). For cancer patients who have received immune checkpoint inhibitor (ICI) therapies, both low EVPL and high ENTPD3 expression indicated a longer post-immunotherapy survival (Figure 5I–K).

### 3.7. EVPL and ENTPD3 Are Expressed in Different Cell Subpopulations in CRC

**Figure 6:**
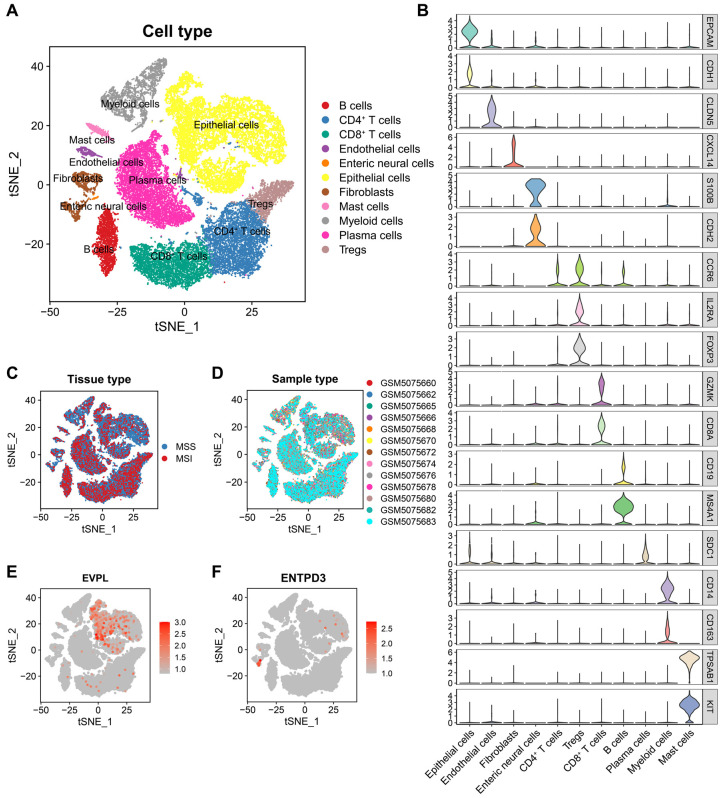
Overview of infiltrating cell types and the expression of EVPL and ENTPD3 in CRC. (**A**) t-SNE plot of 33,495 cells from 13 primary CRC samples (12 MSS samples and 1 MSI sample). (**B**) Violin plot showing the expression of marker genes (Each color represents the expression of a corresponding marker gene in 11 cell subpopulations.). (**C**) t-SNE plot showing cell distribution of MSS CRC and MSI CRC samples. (**D**) t-SNE plot showing cell distribution of CRC samples from different patients. (**E**,**F**) t-SNE plots of the expression of EVPL (**E**) and ENTPD3 (**F**) in 11 cell subpopulations.

After rigorous data quality control, we obtained a total of 33,495 high-quality cells from 13 untreated CRC samples (including 12 MSS samples and 1 MSI sample) using scRNA-seq data of GSE166555. We performed t-distributed Stochastic Neighbor Embedding (t-SNE) downscaling using the “RunTSNE” function (Figure 6A) and then manually annotated each cell subpopulation (Figure 6B). To ensure the maximum elimination of batch effects between samples, we evaluated the distribution of samples with different MSI statuses and patient origins in cell clusters (Figure 6C,D). Similarly, 28,716 cells from 12 matched NAT samples (all exhibiting MSS) were derived and manually annotated (Supplementary Figure S4A). Compared to NATs, the proportion of immune cells in CRC increased, but epithelial cells decreased (Supplementary Figure S4B–F), indicating the activation of anti-tumor immune response. For the two core genes in our study, we found that EVPL was widely expressed in epithelial cells, while ENTPD3 was expressed almost exclusively in specific fibroblasts, whether in NATs (Supplementary Figure S4G,H) or CRC (Figure 6E,F). Quantitative analysis proved that the expression of EVPL was dramatically upregulated in epithelial cells, and ENTPD3 expression was dramatically downregulated in fibroblasts when NATs completed malignant transformation (Supplementary Figure S4I,J).

### 3.8. ENTPD3^+^ CAFs Exhibit High Heterogeneity and Tumor-Suppressive Features Distinct from Classical CAFs

**Figure 7:**
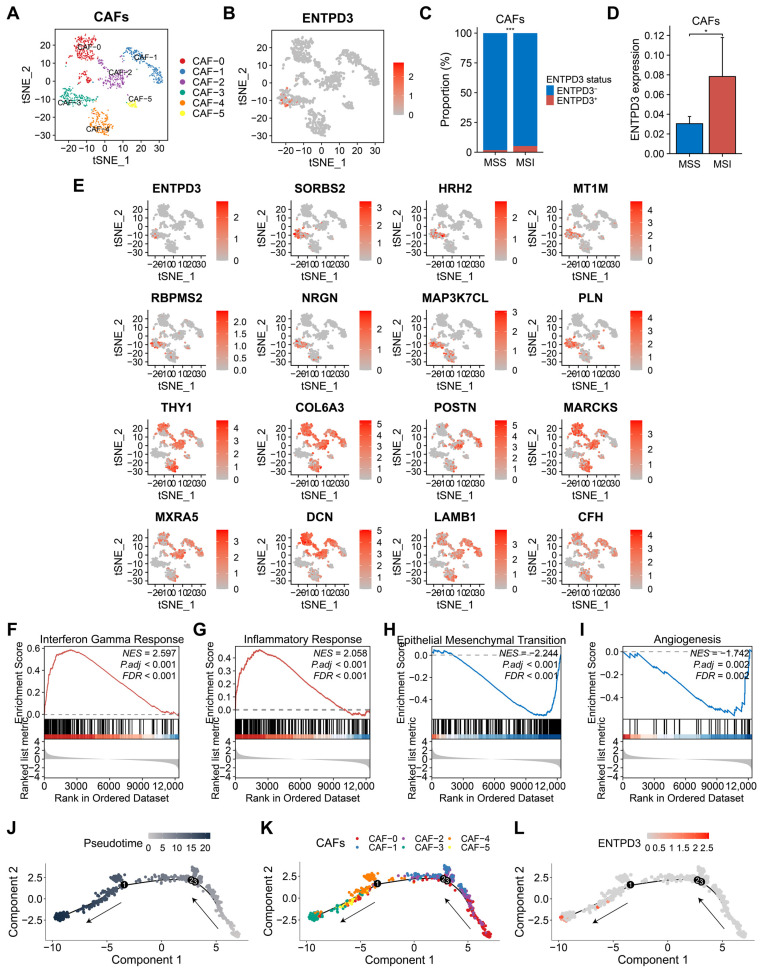
Transcriptional characteristics and heterogeneity of ENTPD3^+^ CAFs in CRC. (**A**) t-SNE plot of 1133 CAFs from 13 primary CRC samples. (**B**) tSNE plot of ENTPD3 expression in CAFs. (**C**) Comparison of the proportion of ENTPD3^+^ CAFs and ENTPD3^−^ CAFs in MSS and MSI CRC. (**D**) Comparison of ENTPD3 expression in MSS and MSI CRC. (**E**) DEGs of the CAF-3 subset. (**F**–**I**) GSEA of ENTPD3^+^ CAFs. (**J**) Trajectory plot of 1133 CAFs in pseudotime. Each point on the pseudotimeline represents a CAF. The lighter the color is, the default starting point is represented, and the darker the color is, the farther it is from the starting point of the pseudotimeline. The direction of the arrows represents the trajectory direction. (**K**) Trajectory plot of 6 CAF subsets in pseudotime. (**L**) Trajectory plot of ENTPD3 expression in 1133 CAFs in pseudotime. * *p* < 0.05 and *** *p* < 0.001.

A total of 1,133 CAFs were clustered into 6 subsets (Figure 7A). Unlike the universal expression of EVPL in epithelial cells, ENTPD3 was expressed almost only in the CAF-3 subset (Figure 7B). We discovered that ENTPD3^+^ CAFs were enriched in MSI CRC, providing an explanation for the association between ENTDP3 and MSI-H status observed in bulk RNA-seq (Figure 7C,D). CAFs, as the most abundant stromal cell component in tumor tissue, have long been known to promote tumor progression through diverse mechanisms [54]. However, recent studies have revealed a high level of heterogeneity and plasticity in CAFs [55], highlighting the desirability to deeply scrutinize the functional differences among distinct CAF subsets. Using the “FindAllMarkers” function, we found that the CAF-3 subset highly expressed several well-recognized tumor suppressor genes such as *SORBS2*, *HRH2*, and *MT1M*, while displaying a low expression of some extracellular matrix-related genes and potential tumor-promoting genes including *COL6A3* and *LAMB1*, which were different from the other 5 CAF subsets (Figure 7E). GSEA indicated that the CAF-3 subset was positively correlated with interferon gamma and inflammatory response, while negatively correlated with epithelial–mesenchymal transition (EMT) and angiogenesis (Figure 7F–I). Thereafter, to understand the differentiation trajectory of CAF subsets, we employed the Monocle 2 tool. The pseudotime analysis demonstrated that the CAF-3 subset represented the terminal stage of CAF differentiation and that ENTPD3^+^ CAFs emerged as the most mature phase within the CAF-3 subset (Figure 7J–L).

### 3.9. ENTPD3^+^ CAFs Are Extraordinarily Sensitive and Specific in Response to Real Effective Immunotherapy

**Figure 8:**
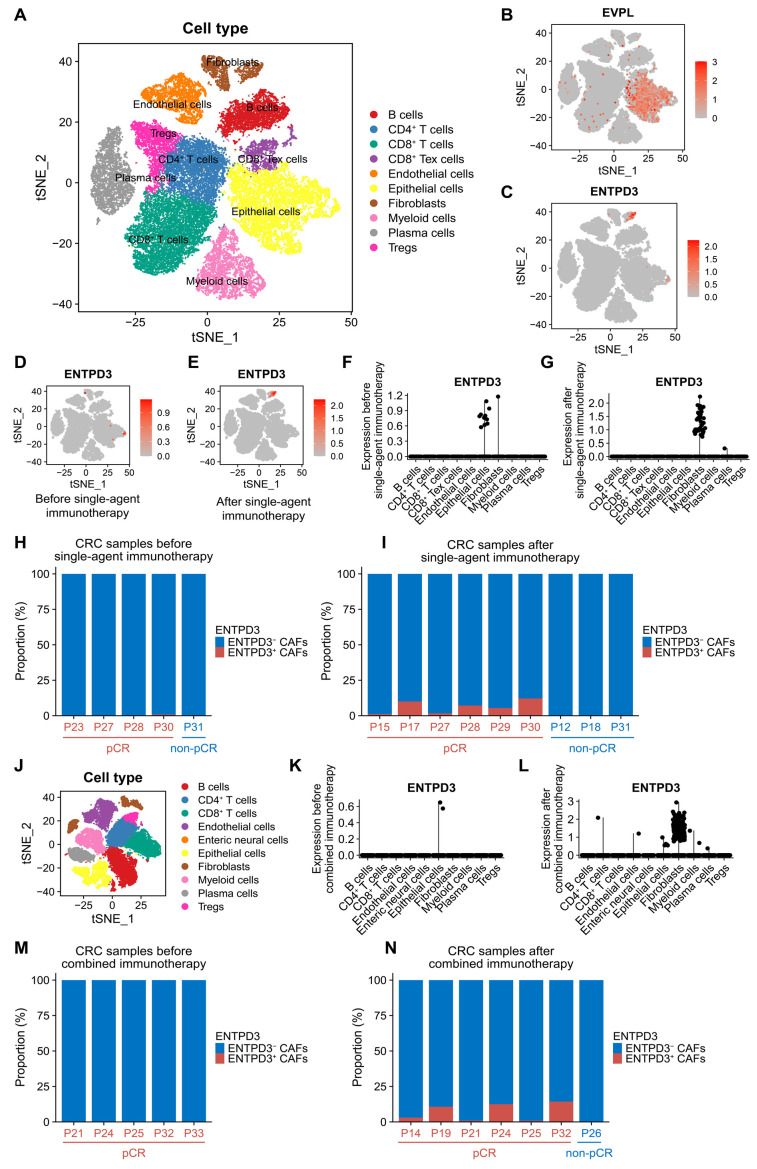
Cell features and global dynamics changes of ENTPD3^+^ CAFs during immunotherapy. (**A**) t-SNE plot of 38,564 cells from 14 primary CRC samples before and after single-agent immunotherapy. (**B**,**C**) tSNE plots of the expression of EVPL (**B**) and ENTPD3 (**C**) in 10 cell subpopulations. (**D**,**E**) tSNE plots of ENTPD3 expression in 10 cell subpopulations before (**D**) and after (**E**) single-agent immunotherapy. (**F**,**G**) Dot plots of ENTPD3 expression in 10 cell subpopulations before (**F**) and after (**G**) single-agent immunotherapy. (**H**,**I**) The proportion of ENTPD3^+^ CAFs in total CAFs before (**H**) and after (**I**) single-agent immunotherapy. (**J**) t-SNE plot of 37,018 cells from 12 primary CRC samples before and after combined immunotherapy. (**K**,**L**) Dot plots of ENTPD3 expression in 10 cell subpopulations before (**K**) and after (**L**) combined immunotherapy. (**M**,**N**) The proportion of ENTPD3^+^ CAFs in total CAFs before (**M**) and after (**M**) combined immunotherapy.

Based on a recent large-scale single-cell sequencing study of MSI-H CRC patients undergoing neoadjuvant PD-1 blockade (NCT03926338/GSE205506), we characterized the cell features and global dynamics of ENTPD3^+^ CAFs in CRC immunotherapy (Figure 8A). The analysis of 38,564 cells from 10 patients in the single-agent immunotherapy cohort revealed widespread expression of EVPL in epithelial cells and evident expression of ENTPD3 in specific CAFs, consistent with our initial finding (Figure 8B,C). For the 5 patients who underwent pre-treatment tumor biopsies, ENTPD3 was observed to be expressed in small amounts in epithelial cells (Figure 8D,E). Among these 5 patients, 4 received subsequent PD-1 blockade, and 3 ultimately achieved pathological complete response (pCR). Interestingly, all 3 patients (P27, P28, P30) who achieved pCR showed a disappearance of ENTPD3^+^ epithelial cells and a clear emergence of ENTPD3^+^ CAFs, while 1 patient (P31) who did not achieve pCR was persistently free of ENTPD3^+^ CAFs. For the 5 patients who only underwent post-treatment tumor biopsies, ENTPD3^+^ CAFs were also obviously present in 3 pCR patients (P15, P17, P29) but completely absent in 2 non-pCR patients (P12, P18) (Figure 8H,I). Next, 9 patients in the combined immunotherapy cohort were treated with both PD-1 blockade and COX-2 inhibition. After excluding one sample with insufficient CAFs (P11′s post-treatment sample contained only 3 CAFs), we obtained 5 pre-treatment and 7 post-treatment samples from the 9 patients (Figure 8J). Similarly, ENTPD3^+^ CAFs were absent in all 5 patients before combined immunotherapy. For the 7 patients after combined immunotherapy, 6 patients who achieved pCR (P14, P19, P21, P24, P25, P32) notably developed ENTPD3^+^ CAFs, while 1 non-pCR patient (P26) showed a continuous deletion, which was identical to the observations in the single-agent immunotherapy cohort (Figure 8M,N). Additionally, effective PD-1 blockade not only sensitively resulted in an increase in the abundance of ENTPD3^+^ CAFs but also significantly upregulated the expression level of ENTPD3 within the CAF subpopulation in both cohorts (Figure 8F,G,K,L). These findings provided further insights into the unique immune phenotype of ENTPD3^+^ CAFs and offered information for future immunotherapy.

### 3.10. Identification of Potential Drugs Targeting EVPL and ENTPD3

**Figure 9:**
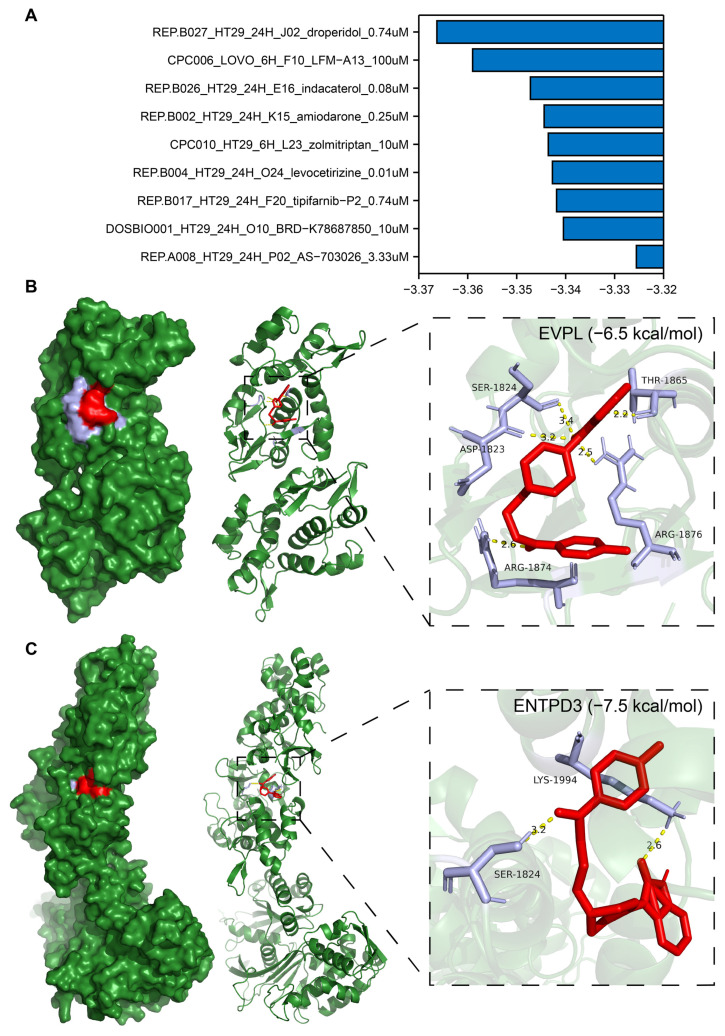
Prediction of drug candidates and binding of droperidol to the core targets EVPL and ENTPD3 using molecular docking analysis. (**A**) Score table for the drugs in the connectivity map database. A z-score greater than 0 indicates upregulation of EVPL and downregulation of ENTPD3 by the drug, and the opposite is true for z-scores less than 0. The larger the absolute value of the z-score, the stronger the drug’s effect. (**B**,**C**) The docking result of droperidol to EVPL (**B**) and ENTPD3 (**C**). Diagram of the droperidol and protein combination.

Using the LINCS, we screened 9 candidate compounds (droperidol, LFM-A13, indacaterol, amiodarone, zolmitriptan, levocetirizine, tipifarnib-P2, BRD-K78687850, and AS-703026; Figure 9A) potentially targeting EVPL and ENTPD3 out of 33,866 small molecular compounds. To predict whether the candidate compounds could serve as direct inhibitors of EVPL and agonists of ENTPD3, we carried out molecular docking utilizing the AutoDock Vina program. Surprisingly, droperidol exhibited high binding stability to both EVPL and ENTPD3 proteins with binding free energies of −6.5 kcal/mol and −7.5 kcal/mol, respectively. The five amino acid residues ASP-1823, SER-1824, THR-1865, ARG-1876, and ARG18741 of EVPL formed tight hydrogen bonds with droperidol (Figure 9B). For ENTPD3, droperidol not only interacted with residues SER-1824 and LYS-1994 through the formation of hydrogen bonds but also bound to a similar pocket region on this protein (Figure 9C). Therefore, droperidol possessed favorable affinity towards both EVPL and ENTPD3 and may be a promising drug for targeting the core genes of T2DM and CRC.

## 4. Discussion

As early as the 1930s, Alexander Marble noted an association between having diabetes mellitus and an increased incidence of several cancers [56]. However, until now, the characteristics of the evident crosstalk based on gene regulation between T2DM and CRC are still obscure. To the best of our knowledge, our study is the first to utilize a comprehensive bioinformatics analysis approach integrating bulk RNA-seq and scRNA-seq to elucidate the common mechanisms and molecular markers between T2DM and CRC.

In our study, by identifying shared genes between key module genes and DEGs, we discovered eight crucial genes with high credibility and finalized the core genes *EVPL* and *ENTPD3* for T2DM and CRC. EVPL is a member of the plakin family that forms the desmosomes and epidermal cornified envelope, localized in the typical esophageal cancer locus on chromosome 17q25 [57]. As a component involved in constituting intercellular junctions, patients with autoimmune mucocutaneous disorder or paraneoplastic pemphigus develop antibodies against its encoded protein [58]. In malignant tumors, the deletion of EVPL is customarily considered to be associated with the formation and radiotherapy resistance of esophageal squamous cell carcinoma [59]. Unfortunately, few studies have focused on the potential role of EVPL in other cancers or chronic diseases. Extracellular nucleotides and nucleosides regulate a variety of physiological functions through purinergic signaling, including immunity, inflammation, thrombosis, tissue repair, and metabolism [60,61]. ENTPD3, a member of the nucleoside triphosphate diphosphohydrolase (NTPDase) family [62], is expressed primarily in brain neurons [63], pancreatic islets of Langerhans [64], and digestive tract epithelium [65]. As a typical membrane-bound enzyme, ENTPD3 hydrolyzes extracellular ATP to ADP/AMP at physiological pH, making it vital for the regulation of extracellular purinergic signaling [66]. Previous studies have well characterized the dysfunction of ENTPD3 in human Parkinson’s disease [67], Alzheimer’s disease [68], and Crohn’s disease [69]. In recent years, the role of ENTPD3 in glucose homeostasis and cancer progression has also been preliminarily explored, identifying it as a marker for human mature pancreatic β-cells and a tumor suppressor in breast cancer [70,71,72]. However, different studies reported positive [71] and irrelevant [70] correlations for ENTPD3 expression and pancreatic β-cell activity. Although ENTPD3 is obviously expressed in both human pancreatic islets and gut, its molecular features and disease relevance in T2DM and CRC are an outstanding and unresolved issue.

Our results demonstrated that EVPL upregulation and ENTPD3 downregulation were intrinsic molecular features shared by both T2DM and CRC and were associated with their malignant progression and adverse prognosis. Enjyoji et al. confirmed that the deletion of ENTPD1 led to impaired glucose tolerance associated with hepatic insulin resistance, which, to some extent, supported our findings on ENTPD3 in T2DM [73], since ENTPD3 is structurally and functionally extraordinarily similar to ENTPD1 and is more abundantly expressed in Langerhans’ islets [74].

Although it has begun to be noticed that T2DM is not only a metabolic disease but also an immune disease [75], previous studies have often only attempted to elucidate the role of metabolic factors in the co-development of T2DM and CRC. Enrichment analysis of genes functionally related to *EVPL* and *ENTPD3* indicated that EVPL might be involved in epithelial cell adhesion and migration, while ENTPD3 was implicated in nucleotide and energy molecule metabolism, which was consistent with their established gene functions. Surprisingly, genes centered on *EVPL* and *ENTPD3* in the network were co-enriched in the regulation of immune cells and immune responses, pointing to the important role that EVPL- and ENTPD3-related immune dysregulation may play in the co-progression of T2DM and CRC. Further analysis revealed that both EVPL upregulation and ENTPD3 downregulation predicted a pro-T2DM (high levels of Th1/Th2 ratio and neutrophils as well as low levels of NK cells) and pro-CRC (overall significantly lower immune cell levels) immune infiltration phenotype. In addition, this aberrant expression represented typical “cold” CRC (low immune cell infiltration, low immune checkpoint expression, and an MSS/MSI-L phenotype) that was unresponsive to immunotherapy. Analysis of multiple other cancers further showed that both high EVPL and low ENTPD3 expression predicts poor response to immunotherapy, confirming the tissue-wide and disease-wide applicability of EVPL- and ENTPD3-associated immune dysregulation.

At the single-cell level, we found that ENTPD3 was expressed exclusively in specific fibroblasts in CRC. Over the years, the adequate characterization of CAFs in promoting cancer initiation and maintenance has rendered them a hugely attractive anti-cancer target, resulting in a plethora of clinical trials focused on CAFs and/or related pathways. However, therapeutic strategies targeting CAFs or related stromal components have yielded little success and even shortened patients’ survival in some cases [76,77]. These unforeseen outcomes underscored the importance of simultaneously considering both the tumor-promoting and tumor-suppressive functions of CAFs. Indeed, CAFs stand as one of the most functionally diverse and hugely plastic cell populations in the TME. By now, the rapid advances of single-cell sequencing technology have made it possible to perform comprehensive, high-resolution gene expression analysis of individual CAF cells, thus facilitating better unraveling of the cellular heterogeneity within the CAF population and allowing for a thorough examination of how these specific features are intricately linked to tumor progression and therapeutic response. Although ENTPD3^+^ CAFs did not reach a dominant abundance in all CRC cases, they were markedly enriched in MSI CRC. Molecular markers and enrichment analysis proved the great heterogeneity within the CAF population in CRC and indicated the potential anti-tumor functions of ENTPD3^+^ CAFs distinct from prototypical tumor-promoting-like CAFs. Pseudotime analysis showed that ENTPD3^+^ CAFs were at the ultimate terminal phase of differentiation among all CAF subsets, prompting that their emergence may signify reactive anti-tumor immunity. Hence, the development of ENTPD3 agonists targeting ENTPD3^+^ CAFs instead of inhibitors is likely a potential strategy for treating CRC.

Immunotherapy has revolutionized the cancer treatment landscape. Unfortunately, despite clear clinical evidence of ICI efficacy in MSI-H CRC, the fact is that more than half of MSI-H CRC patients will not respond to immunotherapy, and there remains a lack of reliable biomarkers and efficacious treatment strategies to guide immunotherapy in MSS CRC [78]. According to our unique findings, ENTPD3^+^ CAFs were not only extremely sensitive but also completely specific in response to truly effective immunotherapy (100% sensitivity and specificity in both cohorts), meaning that MSI-H CRC patients who would benefit from immunotherapy may be well screened by pre-existing ENTPD3^+^ CAFs. Therefore, ENTPD3 was potentially not only a biomarker for identifying MSI-H CRC but also an ideal biomarker for CRC immunotherapy. From a perspective beyond traditional biomarkers, the presence status of ENTPD3^+^ CAFs in CRC patients was likely a natural mapping of the balance between the body’s anti-tumor immune system and the constantly changing tumor immune microenvironment. Digging deeper into the role of the T2DM and CRC core gene *ENTPD3* in CRC anti-tumor immunity would be instrumental in optimizing the clinical values of ICIs in the era of immunotherapy, whether in terms of predictive biomarkers or improving immunotherapy efficiency.

Finally, we predicted the potential for droperidol to be both a novel selective EVPL inhibitor and an ENTPD3 agonist. Droperidol is a butyrophenone derivative and dopamine D2 receptor antagonist conventionally utilized for the amelioration of chemotherapy-associated nausea, vomiting, and postoperative pain in oncological settings and employed as a premedication for anesthesia [79]. However, there is yet no report on its direct therapeutic implications in CRC. More careful investigation into the targeting effects of droperidol on EVPL and ENTPD3 may confer a potential advantage for those CRC patients who fail to benefit from traditional therapies. On the other hand, our study has some limitations. Additional experiments are warranted to further validate the molecular mechanisms of EVPL and ENTPD3. Moreover, the utilization of ENTPD3 as an independent biomarker for CRC immunotherapy needs to be prospectively evaluated in large-sample, multicenter clinical cohorts, especially across different subgroups such as MSS CRC.

## 5. Conclusions

By integrating bulk RNA-seq and scRNA-seq for the first time, our study proves that upregulation of EVPL and downregulation of ENTPD3 are key molecular features of comorbidity of T2DM and CRC. EVPL and ENTPD3 are significantly involved in immunological pathways and are closely associated with the aberrant immune landscapes and altered immune responses of the two diseases, highlighting the importance of focusing on immunological mechanisms in the comorbidity of T2DM and CRC. Additionally, droperidol may serve as a therapeutic drug simultaneously targeting EVPL and ENTPD3, potentially offering survival benefits for CRC patients with concurrent T2DM.

## Data Availability

The original contributions presented in the study are included in the article/Supplementary Materials; further inquiries can be directed to the corresponding authors.

## Supplementary Materials

The following supporting information can be downloaded at: https://www.mdpi.com/article/10.3390/biom14060693/s1, Figure S1: Characterization of the shared DEGs between T2DM and CRC. (A) GO analysis of the shared DEGs. (B) KEGG analysis of the shared DEGs. (C) Venn diagram of the shared DEGs with the same changing trend between T2DM and CRC; Figure S2: Relationship of EVPL and ENTPD3 expression with different clinical and clinicopathological parameters in T2DM and CRC patients. (A) Scatterplot of EVPL and ENTPD3 expression and HbA1c level in T2DM patients. (B) The expression of EVPL and ENTPD3 in CRC tissues with different histologic grades. (C) Comparison of the expression of EVPL and ENTPD3 in normal colorectal mucosa, colorectal adenoma, and colorectal adenocarcinoma. (D) Comparison of the expression of EVPL and ENTPD3 in localized CRC and metastatic CRC. (E) Comparison of the expression of EVPL and ENTPD3 in normal colon, normal liver, primary CRC, and liver metastatic tissue. **p* < 0.05, ***p* < 0.01, and ****p* < 0.001; Figure S3: Gene interaction network and functional enrichment analysis of *EVPL* and *ENTPD3*. (A) Gene interaction network of *EVPL* and *ENTPD3* and their interacting genes. (B) GO analysis of *EVPL* and *ENTPD3* and their interacting genes; Figure S4: Overview of infiltrating cell types and the expression of EVPL and ENTPD3 in NATs. (A) t-SNE plot of 28,716 cells from 12 NAT samples (12 matched MSS samples). (B) The proportion of immune, stromal, and epithelial cells in NATs and CRC. (C–F) Comparison of the proportion of CD8^+^ T cells, (C) T cells, (D) immune cells, (E) and epithelial cells (F) in NATs and CRC. (G, H) t-SNE plots of the expression of EVPL (G) and ENTPD3 (H) in 9 cell subpopulations. (E) Comparison of EVPL expression in epithelial cells of NATs and CRC. (F) Comparison of ENTPD3 expression in fibroblasts of NATs and CRC. **p* < 0.05, ***p* < 0.01, and ****p* < 0.001; Table S1: The details and download sources of T2DM and CRC datasets for the research; Table S2: Detailed information on the inclusion criteria for each T2DM and CRC dataset; Table S3: The details and download sources of immunotherapy datasets for the research; Table S4: Detailed information on the inclusion criteria for each immunotherapy dataset; Table S5: The details and download sources of single-cell datasets for the research; Table S6: Clinical features per patient in the single-cell dataset GSE166555, related to Figure 6 and Figure 7 and Supplementary Figure S4; Table S7: Clinical features per patient in the single-cell dataset GSE205506, related to Figure 8; Table S8: Detailed information on the inclusion criteria for each single-cell dataset.
