# Supplementary material for: Exploring the Molecular Mechanism of Comorbidity of Type 2 Diabetes Mellitus and Colorectal Cancer: Insights from Bulk Omics and Single-Cell Sequencing Validation
Source: Biomolecules. 2024 Jun 14;14(6):693. doi: 10.3390/biom14060693 (PMC11201668; doi:10.3390/biom14060693)
Supplement: Supplementary file 1 [file biomolecules-14-00693-s001.zip › biomolecules-2930513-supplementary.pdf]

biomolecules

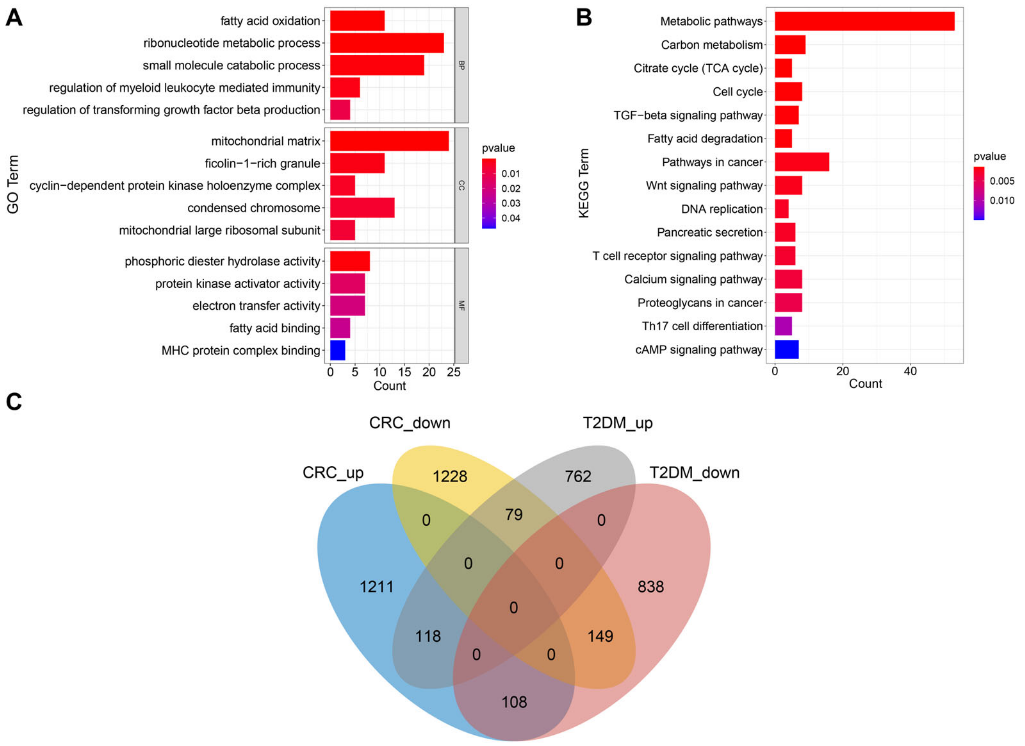

Supplementary Figure S1. Characterization of the shared DEGs between T2DM and CRC. **(A)** GO analysis of the shared DEGs. **(B)** KEGG analysis of the shared DEGs. **(C)** Venn diagram of the shared DEGs with the same changing trend between T2DM and CRC.

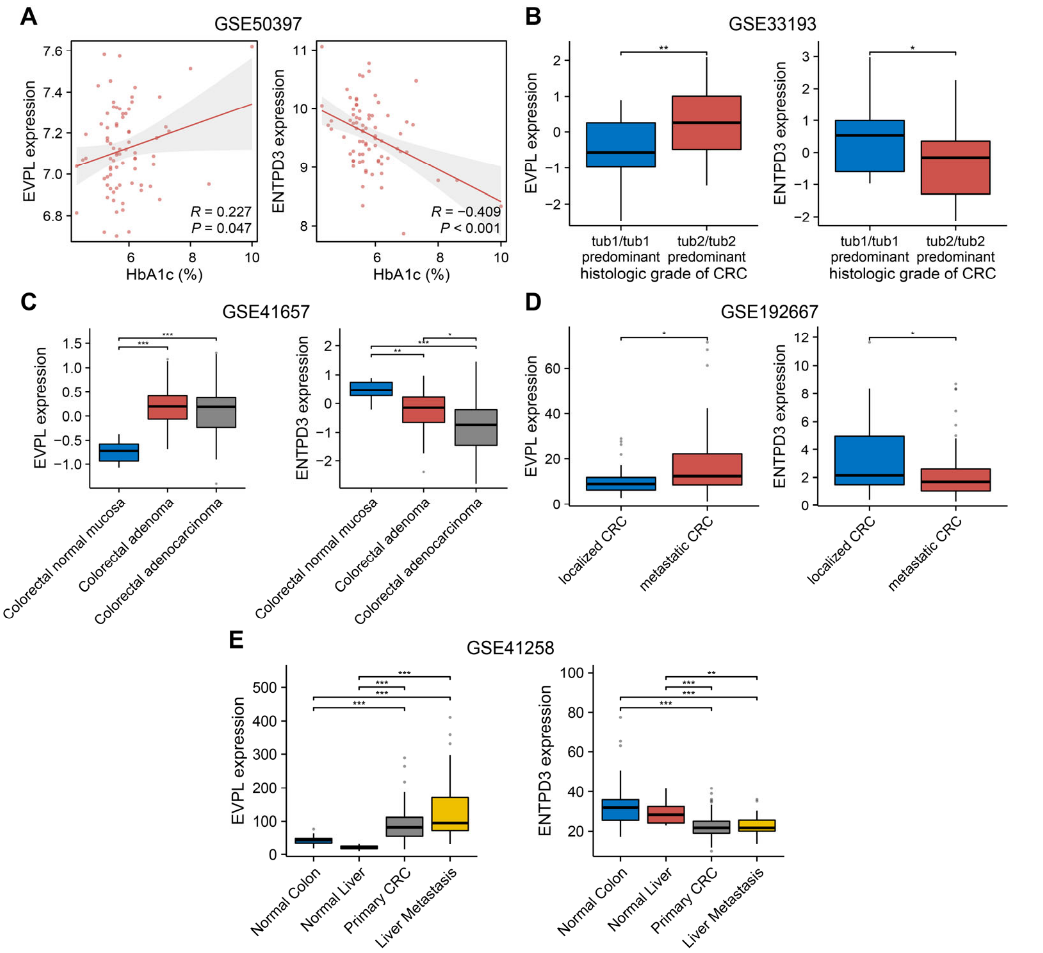

Supplementary Figure S2. Relationship of EVPL and ENTPD3 expression with different clinical and clinicopathological parameters in T2DM and CRC patients. **(A)** Scatterplot of EVPL and ENTPD3 expression and HbA1c level in T2DM patients. **(B)** The expression of EVPL and ENTPD3 in CRC tissues with different histologic grades. **(C)** Comparison of the expression of EVPL and ENTPD3 in normal colorectal mucosa, colorectal adenoma, and colorectal adenocarcinoma. **(D)** Comparison of the expression of EVPL and ENTPD3 in localized CRC and metastatic CRC. **(E)** Comparison of the expression of EVPL and ENTPD3 in normal colon, normal liver, primary CRC, and liver metastatic tissue. *$P < 0.05$, **$P < 0.01$, and ***$P < 0.001$.

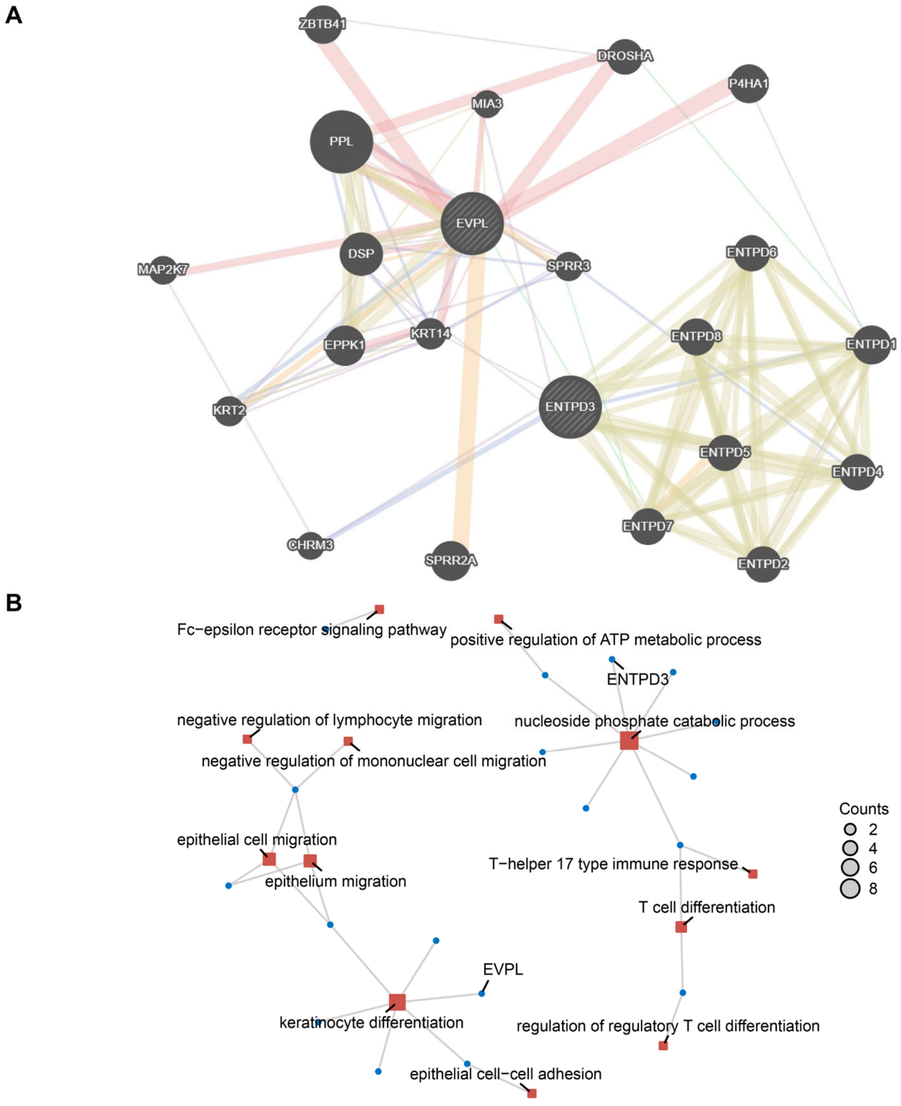

Supplementary Figure S3. PPI network and functional enrichment analysis of EVPL and ENTPD3. **(A)** PPI network of EVPL and ENTPD3 and their interacting proteins. **(B)** GO analysis of EVPL and ENTPD3 and their interacting proteins.

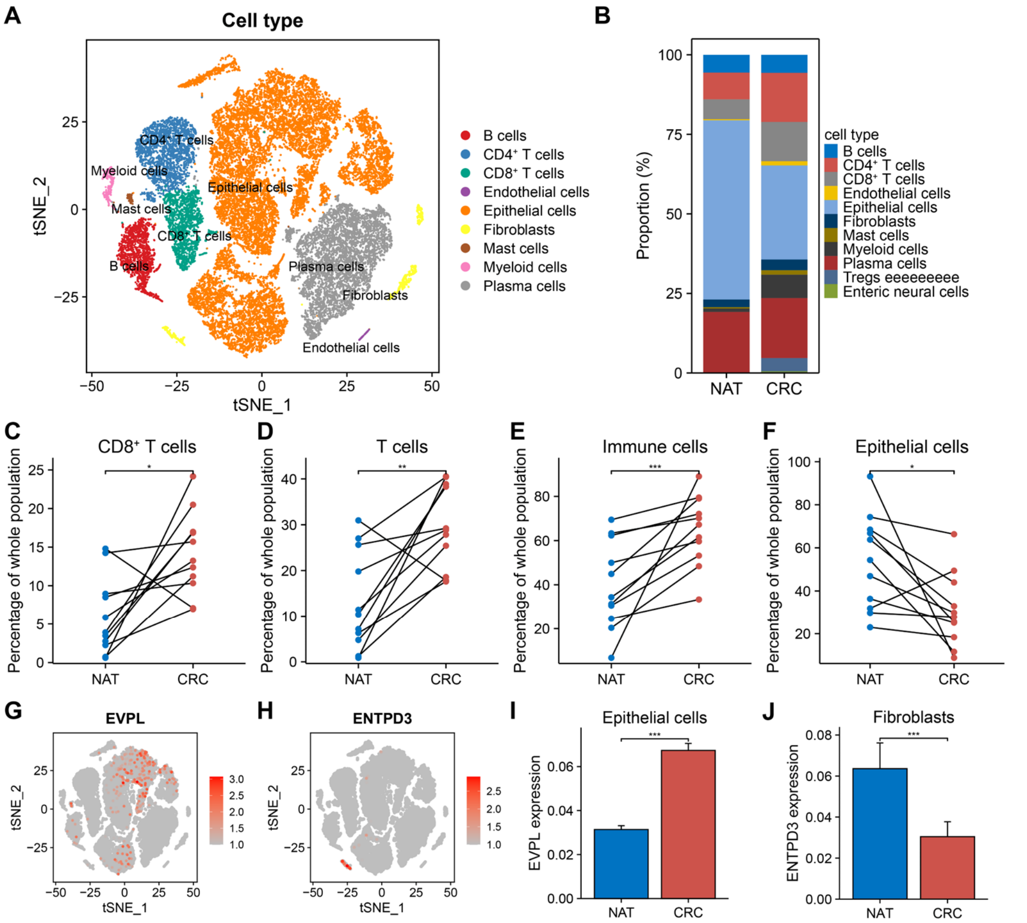

Supplementary Figure S4. Overview of infiltrating cell types and the expression of EVPL and ENTPD3 in NAT. **(A)** t-SNE plot of 28,716 cells from 12 NAT samples (12 matched MSS samples). **(B)** The proportion of immune, stromal, and epithelial cells in NAT and CRC. **(C-F)** Comparison of the proportion of CD8+ T cells, **(C)** T cells, **(D)** immune cells, **(E)** and epithelial cells **(F)** in NAT and CRC. **(G, H)** t-SNE plots of the expression of EVPL **(G)** and ENTPD3 **(H)** in 9 cell clusters. **(E)** Comparison of EVPL expression in epithelial cells of NAT and CRC. **(F)** Comparison of ENTPD3 expression in fibroblasts of NAT and CRC. $^{*}P < 0.05$, $^{**}P < 0.01$, and $^{**}P < 0.001$.

Supplementary Table S1

The details and download sources of T2DM and CRC datasets for the research.

Series Accession Number	Disease	Sample sources	No. of Total Samples	Platform Accession Number	Chip Type	Region	PMID	Group	Download Sources
GSE20966	T2DM	10 patients and 10 controls	20	GPL1352	Affymetrix	United States	20,644,627	WGCNA analysis	the GEO database
GSE25724	T2DM	6 patients and 7 controls	13	GPL96	Affymetrix	Italy	21,127,054	DEG identification	the GEO database
GSE50397	T2DM	89 patients and controls	89	GPL6244	Affymetrix	Sweden	25,015,099; 25,201,977;	Validation	the GEO database

							25,489,054; 30,762,474; 28,637,794; 31,550,518; 33,402,679		
GSE184050	T2DM	25 patients and 33 controls	116	GPL11154	Illumina	United States	35,157,052	Validation	the GEO database
GSE41762	T2DM	20 patients and 54 controls	74	GPL6244	Affymetrix	Sweden	23,140,642; 25,298,321	Validation	the GEO database
GSE32323	CRC	17 patients	34	GPL570	Affymetrix	Japan	22,399,497	WGCNA analysis	the GEO database
GSE68468	CRC	274 patients	378	GPL96	Affymetrix	United States	19,359,472, 12,801,868, 17,699,774, 17,044,051, 16,489,013, 18,483,253, 15,722,375	DEG identification	the GEO database
TCGA-CRC	CRC	622 patients	698	NA	NA	United States	NA	Validation	the TCGA database
GSE25070	CRC	26 patients	52	GPL6883	Illumina	United States	21,659,424	Validation	the GEO database
GSE113513	CRC	14 patients	28	GPL15207	Affymetrix	China	35,096,810	Validation	the GEO database
GSE24549	CRC	83 patients	166	GPL5175	Affymetrix	Norway	22,213,796	Validation	the GEO database
GSE143985	CRC	85 patients	91	GPL570	Affymetrix	Japan	32,328,985	Validation	the GEO database
GSE33193	CRC	100 patients	100	GPL6480	Agilent	Japan	NA	Validation	the GEO database
GSE41657	CRC	25 patients and 63 controls	88	GPL6480	Agilent	China	NA	Validation	the GEO database
GSE192667	CRC	89 patients	89	GPL20301	Illumina	Japan	35,128,352	Validation	the GEO database
GSE41258	CRC	274 patients	378	GPL96	Affymetrix	Israel	19,359,472; 28,958,617	Validation	the GEO database

Supplementary Table S2.

Detailed information on the inclusion criteria for each T2DM and CRC dataset.

GSE	Detailed information
**GSE20966**	The dataset contains gene expression profiles of beta-cells obtained by laser capture microdissection technique from the cadaveric pancreases of 10 type 2 diabetic subjects and 10 control subjects.
**GSE25724**	The dataset contains human islet gene expression profiles obtained by collagenase digestion and density gradient purification from 6 type 2 diabetic subjects and 7 non-diabetic subjects.
**GSE50397**	The dataset contains human islet gene expression profiles obtained from 89 subjects with different levels of glycosylated hemoglobin (HbA1c).
**GSE184050**	The dataset contains longitudinal whole blood gene expression profiles of 25 type 2 diabetic subjects and 33 healthy controls.
**GSE41762**	This dataset contains human islet gene expression profiles obtained from 20 type 2 diabetic subjects and 57 non-diabetic subjects.
**GSE32323**	The dataset contains gene expression profiles of cancer and matched non-cancerous tissues from 17 colorectal cancer patients. The dataset also provides gene expression profiles of five colorectal cancer cell lines (COLO320, HCT116, HT29, RKO, SW480) treated or not with 5-aza-2'-deoxycytidine.
**GSE68468**	

	The dataset contains gene expression profiles of primary cancer, liver metastasis, lung metastasis, high grade dysplasia, polyp, microadenoma, normal colon mucosa, normal liver, and normal lung tissues from 261 colon cancer patients, as well as gene expression profiles of polyp and normal colon mucosa tissues from 13 colon polyp patients. In addition, the dataset also provides gene expression profiles of 7 colon cancer cell lines (DLD-1, HT29, HTB39, SW1116, WiDr, SW620, and LoVo), 1 normal colonic epithelial cell line (NCM460), 1 breast cancer cell line (MCF7), and 1 prostate adenocarcinoma cell line (LNCaP).
**TCGA-CRC**	The dataset contains gene expression profiles of cancer and matched non-cancerous tissues from 622 colorectal cancer patients. Additionally, the dataset provides comprehensive clinical information for research on genetic variations, biomarkers, and various clinicopathological features associated with colorectal cancer. This information includes:<br>1. basic patient information: gender, age, race, and ethnicity;<br>2. disease diagnosis: year of diagnosis, initial disease stage (stage I, II, III, IV), pathological stage (TNM stage), and pathological type.<br>3. treatment history: details of surgical treatment (such as type of surgery and date), radiotherapy treatment details, chemotherapy treatment details, and targeted therapy details.<br>4. follow-up and survival information:<br>survival status (alive or deceased), survival time, and disease recurrence status.<br>5. biosample-related information: types of samples obtained (tumor tissue and normal tissue), sample processing and preservation methods, types of molecular analysis data (such as whole-genome sequencing and RNA sequencing, etc.)

	6. lifestyle and risk factors: smoking history, alcohol consumption history, and family history of genetic disorders<br>7. other relevant clinical information:<br>body mass index (BMI) and other relevant health conditions or comorbidities.
**GSE25070**	The dataset contains gene expression profiles of cancer and matched histologically normal adjacent colonic tissues from 26 colorectal cancer patients.
**GSE113513**	The dataset contains gene expression profiles of cancer and matched non-cancerous tissues (at least 5 cm away) from 14 colorectal cancer patients who had undergone surgical resection of colorectal cancer. No patients had received radio- or chemotherapy prior to surgery.
**GSE24549**	The dataset contains genome-wide expression at the exon level in colorectal cancer tissues from 83 colorectal cancer patients, with disease-free survival status and disease-free survival time provided in full for each patient. None of the patients had received adjuvant chemotherapy, which was introduced as standard treatment for patients with stage III CRC aged <75 years in Norway in 1997.
**GSE143985**	The dataset contains gene expression profiles of primary colorectal cancer tissues from 85 colorectal cancer patients, with disease-free survival status and disease-free survival time provided in full for each patient.
**GSE33193**	The dataset contains gene expression profiles of colorectal cancer tissues from 100 patients with

	unresectable and advanced or recurrent colorectal cancer who underwent surgical resection between 1998 and 2010.
**GSE41657**	The dataset contains gene expression profiles of normal colorectal mucosa tissues from 12 hemorrhoid patients, colorectal adenoma tissues from 51 colorectal adenoma patients, and colorectal cancer tissues from 25 colorectal cancer patients.
**GSE192667**	The dataset contains gene expression profiles of colorectal cancer tissues from 89 colorectal cancer patients, 66 of whom had stage IV colorectal cancer.
**GSE41258**	The dataset contains gene expression profiles of biological specimens (including primary colon adenocarcinomas, adenomas, metastasis, and corresponding normal mucosae) from 275 patients who presented at Memorial Sloan-Kettering Cancer Center with a colonic neoplasm between 1992 and 2004.

Note: All datasets are in compliance with GEO's upload regulations.

Supplementary Table S3

The details and download sources of immunotherapy datasets for the research.

Series Accession Number	Cancer Type	Therapy	Drug	No. of Total Samples with Available Clinical Data before Receiving Immunotherapy	Platform	Region	PMID	Download Sources

Gide et al. (anti-PD-1)	Advanced melanoma	Anti-PD-1	Nivolumab/Pembrolizumab	41	Illumina Hiseq 2500	Australia	30,753,825	the TIDE database
Kim et al. (anti-PD-1)	Metastatic gastric cancer	Anti-PD-1	Pembrolizumab	45	NA	South Korea	30,013,197	the TIDE database
IMvigor210 (anti-PD-L1)	Metastatic urothelial cancer	Anti-PD-L1	Atezolizumab	348	Illumina RNAseq	United States	29,443,960	R package "IMvigor210CoreBiologies" downloaded from the article (https://www.nature.com/articles/nature25501)
Braun et al. (anti-PD-1)	Advanced clear cell renal cell carcinoma	Anti-PD-1	Nivolumab	181	NA	United States	32,472,114	Supplementary material in the article (https://www.nature.com/articles/s41591-020-0839-y)
Gide et al. (anti-PD-1+anti-CTLA-4)	Advanced melanoma	Anti-PD-1+anti-CTLA-4	Ipilimumab + Nivolumab/Ipilimumab + Pembrolizumab	32	Illumina Hiseq 2500	Australia	30,753,825	the TIDE database

Note: the Tumor Immune Dysfunction and Exclusion (TIDE) database (http://tide.dfci.harvard.edu/)

Supplementary Table S4

Detailed information on the inclusion criteria for each immunotherapy dataset.

**Dataset**	**Detailed information**
**Gide et al. (anti-PD-1)**	In this cohort, 54 melanoma patients were treated with anti-PD-1 monotherapy (n=12 nivolumab 3 mg/kg 2-weekly; n=42 pembrolizumab 2 mg/kg 3-weekly).<br>41 pre-treatment (PRE) (n=22 responders; n=19 non-responders) and 9 early during treatment (EDT) (n=5 responders; n=4 non-responders) biopsy samples underwent RNA sequencing, with efficacy evaluations result, overall survival

	status, and overall survival time provided in full for each patient.
**Kim et al. (anti-PD-1)**	In this cohort, 61 patients with measurable, histologically confirmed metastatic and/or recurrent gastric adenocarcinomas were treated with anti-PD-1 monotherapy (pembrolizumab 200 mg was administered as a 30-minute intravenous infusion every 3 weeks until documented disease progression).<br>To be eligible to participate in this study, patients were required to meet the following criteria:<br>(1) histologically confirmed diagnosis of gastric or gastroesophageal junctional adenocarcinoma.<br>(2) age of at least 19 years.<br>(3) previous failure of at least 1 line of chemotherapy that included platinum/fluoropyrimidine.<br>(4) willingness to undergo a procedure to obtain fresh-frozen tissue within 42 days of treatment initiation for biomarker analysis.<br>(5) adequate organ function per protocol. (6) at least 1 measurable lesion according to RECIST 1.124, and (7) Eastern Cooperative Oncology Group performance status of 0 or 1. All patients were naive to anti-PD-1, anti-PD-L1, or anti-PD-L2 antibodies.<br>60 patients underwent PRE biopsy (46 stomach/primary tumor, 8 liver, 4 peritoneal, 1 lung, 1 distant lymph node, and 1 paravertebral mass) before study entry, and 57 patients received efficacy evaluations. Ultimately, 45 specimens were of sufficiently high quality for RNA sequencing.
**IMvigor210 (anti-PD-L1)**	In this cohort, RNA was extracted from formalin-fixed and paraffin embedded tumors of 348 bladder cancer patients before treatment with anti-PD-L1 monotherapy (atezolizumab). RNA was sequenced using a capture-based approach

	(exome capture, RNA access), with efficacy evaluation results provided in full for each patient.
**Braun et al. (anti-PD-1)**	In this cohort, 3 prospective clinical trials of anti-PD-1 monotherapy (nivolumab) in advanced clear cell renal cell carcinoma (ccRCC) were included:<br>(1) CheckMate 009 (CM-009; NCT01358721): This prospective clinical trial enrolled 119 patients with metastatic clear cell renal cell carcinoma. To be eligible for the previously treated groups, patients must have been treated with between one and three previous systemic therapies for RCC, with progression following the most recent therapy within 6 months of study enrollment. For the treatment-naïve group, patients must not have received any previous systemic therapy in the metastatic or adjuvant setting. Previously treated patients were randomized 1:1:1 to receive nivolumab 0.3, 2, or 10 mg/kg; treatment-naïve patients received nivolumab 10 mg/kg. Nivolumab was administered as an intravenous infusion on day 1 of the treatment cycle every 3 weeks until confirmed complete response, progressive disease, intolerable adverse events (AEs), or withdrawal of consent.<br>(2) CheckMate 010 (CM-010; NCT01354431): This prospective clinical trial enrolled 168 patients with progressive, advanced/metastatic clear cell renal cell carcinoma who have received prior anti-angiogenic therapy. Patients were randomly assigned (blinded ratio of 1:1:1) to nivolumab 0.3, 2, or 10 mg/kg intravenously once every 3 weeks, until progressive disease (PD), toxicity or discontinue for other reasons.<br>(3) CheckMate 025 (CM-025; NCT01668784): This prospective clinical trial enrolled 821 patients with advanced or metastatic clear cell renal cell carcinoma who have received prior anti-angiogenic therapy. Patients were randomly

	assigned to nivolumab (3 mg/kg solution intravenously every 2 weeks until documented disease progression, discontinuation due to toxicity, withdrawal of consent or the study ends) or everolimus (10 mg tablets by mouth daily until documented disease progression, discontinuation due to toxicity, withdrawal of consent or the study ends)<br>RNA sequencing was performed on biopsy samples from 311 patients before treatment with nivolumab (n=181) or everolimus (n=130), with efficacy evaluations results, progression-free survival status, and progression-free survival time provided in full for each patient.
**Gide et al. (anti-PD-1+anti-CTLA-4)**	In this cohort, 51 melanoma patients were treated with combined ipilimumab and anti-PD-1 immunotherapy (n=11 ipilimumab 3 mg/kg + nivolumab 1 mg/kg; n=40 ipilimumab 1 mg/kg + pembrolizumab 2 mg/kg).<br>32 PRE (n=24 responders; n=8 non-responders) and 9 EDT (n=6 responders; n=3 non-responders) biopsy samples underwent RNA sequencing, with overall survival status and overall survival time provided in full for each patient.

Supplementary Table S5

The details and download sources of single-cell datasets for the research.

Series Accession Number	Disease	Therapy	Drug	No. of Patients	Status at Biopsy	Sample Type	No. of Total Samples	Platform Accession Number	Platform Type	Region	PMID	Download Sources
GSE166555	CRC	-	-	12	All patients were treatment naive.	13 primary CRC and 12 matched normal adjacent tissues	25	GPL23177	10x Genomics	Germany	34,409,732	the GEO database

GSE205506	CRC	Anti-PD-1/anti-PD-1+COX-2 inhibition.	Toripalimab/Toripalimab + Celecoxib	19	For the 10 patients who received anti-PD-1 immunotherapy, 5 patients received a biopsy of the tumor site before treatment and 9 patients received a biopsy of the tumor site after treatment. For the 9 patients who received combined immunotherapy, 5 patients received a biopsy of the tumor site before treatment and 8 patients received a biopsy of the tumor site after treatment.	10 primary CRC before immunotherapy and 17 primary CRC after primary immunotherapy	27	GPL24676 and GPL29480	10x Genomics	China	37,172,580	the GEO database

Supplementary Table S6

Clinical features per patient in the single-cell dataset GSE166555, related to Figure 6-7 and Supplementary Figure 4

Patient ID	Gender	Key Driver Mutations	TNM Stage	Grade	Microsatellite status	Localisation	Inferred progression	Tissues used
P007	M	BRAFV600E, TP53R175H	T2N1b	G3	MSS	C	S	N/T
P008	M	TP53R175H	T4aN2b	G3	MSS	C	I	N/T
P009	M	APCLOH, FBXW7D480H, TP53R248Q	T2N0	G2	MSS	S	C	2N/2T
P012	M	NA	TisN0	G2	MSS	T	?	N/T
P013	M	APCLOH, NOTCH1H1601L	T4aN1bM1	G2	MSS	A	C	N/T
P014	F	APCLOH, 1556insN, BRAFV600E, HRASA59T, PIK3CAE545K, TGFBR2D547H	T3N2b	G3	MSS	A	?	N/T

P016	M	APCLOH, 1396delF, KRASG13C, PIK3CAE545K, FBXW7R278*	T3N0	G2	NA (P016 was described as having MSS CRC in the results section of the article.)	R	C	N/T
P017	F	APCLOH, KRASG13D	T3N2b	G3	MSS	A	C	N/T
P020	M	BRAFV600E, TP53R282W	T2N1a	G2	MSS	R	S	N/T
P021	F	APCLOH, KRASA146T, DNMT1R1384Q	T2N0	G2	MSS	D	C	N/T
P025	M	APCLOH, KRASG13D, TP53G245S	T3N0	G2	MSS	A	C	N/T
P026	M	BRAFV600E, TP53S90fs, AXIN2LOH, PI4KAA1198T	T1N0	G2	MSI	C	S	T

Supplementary Table S7

Clinical features per patient in the single-cell dataset GSE205506, related to Figure 8

Treatment	Patient ID	Tumor anatomical location	Mismatch repair defective protein	Microsatellite status	TNM Stage	Gender	Age (years)	Tumor	
								pre-treatment	Post-treatment
Anti-PD-1	P12	Hepatic flexure of colon	MLH1, PMS2	MSI-H	cT4aN0M0-IIB	M	67	N	Y
	P15	Ascending colon	MSH6	NA	cT4aN1M0-IIIB	F	58	N	Y
	P17	Descending colon	PMS2	MSI-H	cT4aN1M0-IIIB	M	62	N	Y
	P18	Transverse colon	MLH1, PMS2	NA	cT4bN2M0-IIIC	F	31	N	Y
	P23	Sigmoid colon	MSH6	MSI-H	cT4aN2M0-IIIC	M	38	Y	N
	P27	Transverse colon	PMS2	NA	cT4bN2M0-IIIC	F	67	Y	Y
	P28	Sigmoid colon	MSH2, MSH6	NA	cT4bN2M0-IIIC	M	57	Y	Y
	P29	Ascending colon	MSH2, MSH6	NA	cT4bN2M0-IIIC	M	36	N	Y
	P30	Ascending colon	MSH2	MSI-H	cT3N2M0-IIIB	M	45	Y	Y
	P31	Hepatic flexure of colon	MSH2, MSH6	MSI-H	cT4aN2M0-IIIC	M	53	Y	Y

Anti-PD-1+celecoxib	P11	Hepatic flexure of colon	MLH1, PMS2	MSI-H	cT4aN1M0-IIIB	M	65	N	Y
	P14	Transverse colon	MSH6	MSI-H	cT4aN2M0-IIIC	M	50	N	Y
	P19	Sigmoid colon	MLH1, PMS2	NA	cT4aN2M0-IIIC	M	26	N	Y
	P21	Rectum	MLH1, PMS2	NA	cT3N1M0-IIIB	F	52	Y	Y
	P24	Ascending colon	PMS2	MSI-H	cT4aN2M0-IIIC	F	45	Y	Y
	P25	Ascending colon	PMS2	MSI-H	cT4bN2M0-IIIC	F	69	Y	Y
	P26	Hepatic flexure of colon	PMS2	NA	cT3N2M0-IIIB	F	45	N	Y
	P32	Transverse colon	MSH2	MSI-H	cT4bN2M0-IIIC	M	33	Y	Y
	P33	Rectum	MSH2	MSI-H	cT4bN2M0-IIIC	M	37	Y	N

Note: N, Not available; Y, Yes

pCR, pathological complete response; non-pCR, non pathological complete response

Supplementary Table S8

Detailed information on the inclusion criteria for each single-cell dataset.

GSE	Detailed information
**GSE166555**	In this cohort, tissue of 12 patients was collected from tumor areas and adjacent normal tissue after surgical removal of the tumor. Specifically, to capture the diversity of CRC cell states compared to the normal colon epithelium, single-cell transcriptome analysis was performed on twelve previously untreated CRC patients undergoing primary surgery. The investigation included tissue samples encompassing the invasive tumor front and matched non-cancerous tissues. The tumors ranged from stages pTis (Tumor in situ) to pT4, presenting with or without metastasis and situated at various points along the cephalocaudal axis of the colon. Genetic analysis identified mutational patterns indicative of canonical CRC

	progression in most tumors; however, tumors from patients P007, P014, P020, and P026 exhibited the BRAFV600E mutation, frequently associated with the serrated progression pathway, and tumor P008 was linked to colitis. Out of the cohort, eleven tumors were classified as microsatellite-stable (MSS) CRC, whereas the tumor from patient P026 showed microsatellite instability (MSI).
**GSE205506**	In this cohort, single-cell RNA sequencing (scRNA-seq) was conducted on 40 samples of tumor and adjacent normal tissues from 19 patients to delineate the cellular and molecular profiles of immune and stromal cells, as well as dynamics during ICI treatment. patients were from a randomized phase 2 study (NCT03926338) featuring individuals with locally advanced primary invasive d-MMR/MSI-H carcinoma of the colon and rectum, receiving toripalimab with or without celecoxib for six cycles before curative surgical resection (PICC study). A majority of the cases (15 of 19) reached a pathological complete response (pCR), with no residual tumor observable post-neoadjuvant ICI therapy.
