# Supplementary figures and images for: Exploring the Molecular Mechanism of Comorbidity of Type 2 Diabetes Mellitus and Colorectal Cancer: Insights from Bulk Omics and Single-Cell Sequencing Validation
Source: Biomolecules. 2024 Jun 14;14(6):693. doi: 10.3390/biom14060693 (PMC11201668; doi:10.3390/biom14060693)

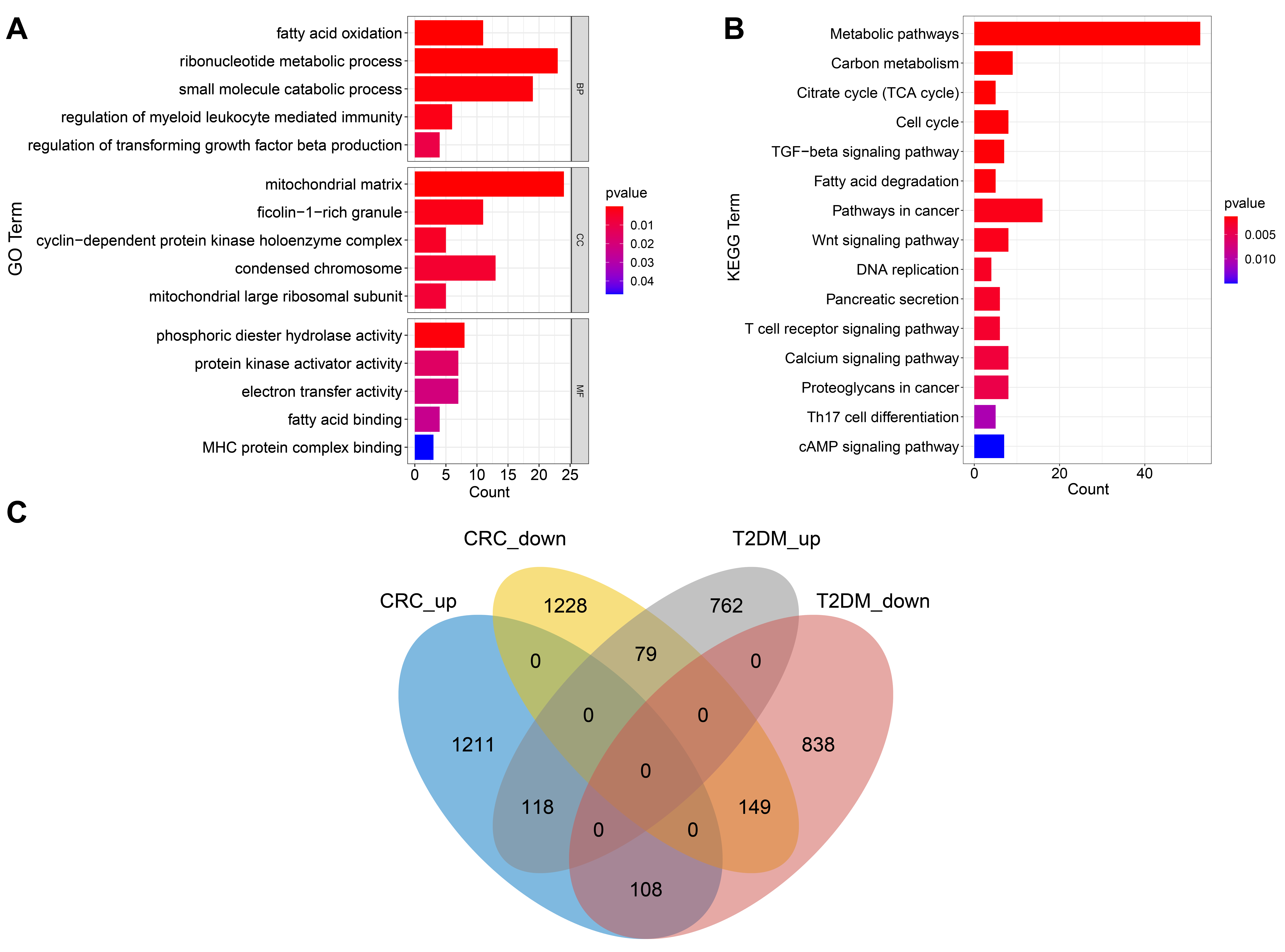

Supplement: Supplementary file 1 [file biomolecules-14-00693-s001.zip › Figure S1-4/Figure S1.tif]
